# Enhancing the statistical probability factor in triplet–triplet annihilation photon upconversion *via* TIPS functionalization

**DOI:** 10.1039/d5sc05248c

**Published:** 2025-10-07

**Authors:** Lukas Naimovičius, Manvydas Dapkevičius, Edvinas Radiunas, Mila Miroshnichenko, Gediminas Kreiza, Carles Alcaide, Paulius Baronas, Yoichi Sasaki, Nobuhiro Yanai, Nobuo Kimizuka, Andrew B. Pun, Miquel Solà, Pankaj Bharmoria, Karolis Kazlauskas, Kasper Moth-Poulsen

**Affiliations:** a Institute of Materials Science of Barcelona (ICMAB-CSIC), Universitat Autònoma de Barcelona Bellaterra Barcelona 08193 Spain kasper.moth-poulsen@upc.edu pbharmoria@icmab.es; b Institute of Photonics and Nanotechnology, Vilnius University Saulėtekio av. 3 LT-10257 Vilnius Lithuania; c Department of Chemistry and Biochemistry, University of California San Diego 92093 La Jolla CA USA; d Institute of Computational Chemistry and Catalysis (IQCC) and Department of Chemistry, Universitat de Girona M. Aurèlia Capmany 69 17003 Girona Spain; e Department of Chemical Engineering, Universitat Politècnica de Catalunya EEBE Eduard Maristany 10–14 08019 Barcelona Spain; f Department of Applied Chemistry, Graduate School of Engineering Kyushu University 744 Moto-oka Nishi-ku Fukuoka 819-0395 Japan; g Catalan Institution for Research & Advanced Studies (ICREA) Pg. Lluís Companys 23 Barcelona Spain; h Department of Chemistry and Chemical Engineering, Chalmers University of Technology Kemivagen 4 Gothenburg 412 96 Sweden

## Abstract

We investigated the influence of triisopropylsilyl (TIPS) functionalization on annihilators in triplet–triplet annihilation photon upconversion, specifically focusing on their spin statistical probability factor. A new green-emitting annihilator 3,9-bis((triisopropylsilyl)ethynyl)perylene (TIPS-PY) displaying a record red-to-green TTA-UC quantum yield of 13.7% (50% theoretical maximum) was synthesized. This remarkable efficiency was achieved due to the following features of the TIPS functionalization of PY: (1) retaining a high fluorescence quantum yield of 95%, (2) reduced triplet energy to 1.29 eV enabling efficient triplet energy transfer (∼100%) from the sensitizer PdTPBP (T_1_ = 1.55 eV), and (3) a high efficiency of singlet generation after triplet coupling, indicated by the statistical probability factor, *f* = 39.2% ± 2.4%. Notably, the *f* value of TIPS-PY surpasses other annihilators in the 470–570 nm emission range. Excited state computational analysis using TheoDORE revealed a higher percentage of charge transfer character in S_0_S_1_ in TIPS-PY compared to PY, indicative of higher singlet-like character in their triplet-pair state ^1^(T_1_T_1_), which can enhance the coupling of the triplet-pair state with the excited singlet-state, thereby increasing the efficiency of singlet generation, a phenomenon undisclosed before. Furthermore, the suitable T_1_ of TIPS-PY enables upconversion of 730 nm light when sensitized with Os(m-peptpy)_2_(TFSI)_2_ (T_1_ = 1.63 eV), demonstrating the broad upconversion range of TIPS-PY in the phototherapeutic window desired for biological applications.

## Abstract 2

A new green-emitting annihilator, TIPS-PY is synthesized, which exhibits the high TTA-UC quantum yield of 13.7% for red-to-green upconversion due to the enhancement of spin statistical probability factor by TIPS functionalization of perylene.

## Graphical abstract

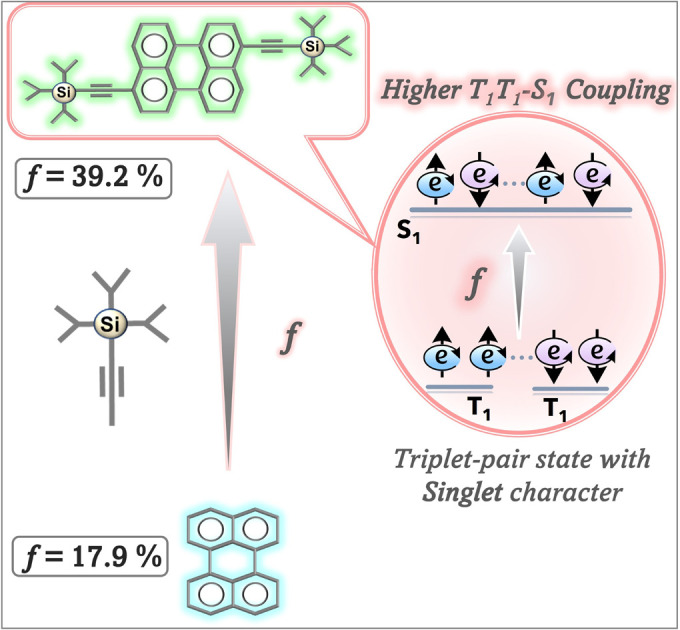

## Introduction

**Scheme 1:**
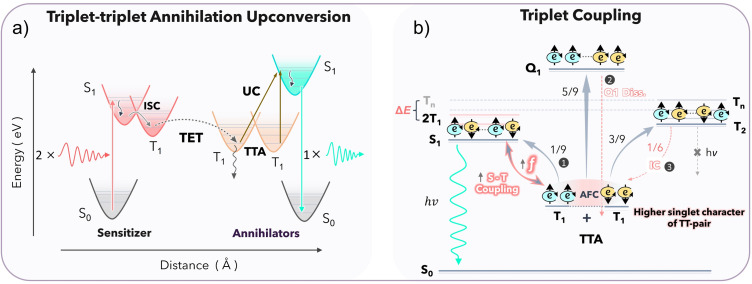
(a) Scheme of TTA-UC indicating conversion of two low-energy photons into one high-energy photon through a series of energy transfer processes. ISC – intersystem crossing, TET – triplet energy transfer, TTA – triplet–triplet annihilation and UC – upconversion (b) schematic illustration of the post-TTA events resulting in the formation of the TT pair with singlet (S_1_, *f* = 1/9), triplet (T_2_, 3/9) and quintet (Q_1_, 5/9) states due to the anti-ferromagnetic coupling (AFC) between triplet-pairs. Further recycling *via* quintet dissociation (Q1 diss.) or internal conversion (IC) can increase the *f* of S_1_ formation to 1/2. A higher singlet character of the TT pair increases the coupling between the TT pair and the singlet state, which further increases the *f*.

Triplet–triplet annihilation photon upconversion (TTA-UC) is a molecular nonlinear optical process that converts two photons with low-energy into one photon with high energy (Scheme 1a).^1^ This phenomenon is attractive compared to other UC processes^2^ due to its operation under incoherent low energy density excitations^3^ which opens many potential applications such as photocatalysis, biological photoactivation, 3D printing, and photovoltaics.^4–11^ A typical TTA-UC system consists of a sensitizer and an annihilator ensemble. The sensitizer absorbs low-energy photons and generates triplet states *via* intersystem crossing (ISC). The annihilator accumulates the triplets through Dexter triplet energy transfer (TET) from the sensitizer and undergoes TTA-UC, generating a photon-emitting high-energy singlet state (Scheme 1a). The efficiency of singlet generation is evaluated by TTA-UC quantum yield (*ϕ*_UC_), which is the product of all operational processes within the TTA-UC system (eqn (1)) and the spin-statistical probability factor (*f*). The *f* determines the probability of singlet generation after triplet coupling (Scheme 1b).1
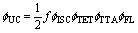
In the eqn (1), *ϕ*_UC_, *ϕ*_ISC_, *ϕ*_TET_, *ϕ*_TTA_, *ϕ*_FL_ represent the quantum yields of upconversion (UC), intersystem crossing (ISC), triplet energy transfer (TET), triplet–triplet annihilation (TTA), and annihilator fluorescence (FL), respectively.

The *f* plays an essential role in TTA-UC by defining the maximum achievable *ϕ*^∞^_UC_ as *f*/2 when all other processes approach unity. As follows, the *f* allows us to assess the intrinsic potential of the annihilator triplets to generate an emissive singlet state (Scheme 1b). It can be altered by suitable molecular engineering of annihilator chromophores to control the triplet coupling strength, which has been investigated in this work. The TTA results in the formation of a triplet-pair (T_1_⋯T_1_) whose net spin can be S = 0, 1, or 2, hence possessing singlet, triplet, or quintet character. According to the adapted Merrifield model including exchange interactions (J), under zero-field splitting, coupling results in the formation of 9 spin eigenstates of T_1_T_1_ pairs with an overall fraction of 1/9, 3/9, and 5/9 of singlet, triplet, and quintet pair states (Scheme 1b). Triplet coupling can be expressed simply by Heisenberg's spin-only Hamiltonian (Ĥ) using eqn 2

2 Ĥ = −2JŜ_1_·Ŝ_2_

where Ŝ_1_ and Ŝ_2_ are individual spin operators of the two individual interacting triplets, and *J* is the magnetic exchange parameter that also defines the strength of inter-triplet exchange interactions.^1,5^ In the case of strong electronic coupling, the quintet state (Q_1_) is energetically inaccessible and cannot form the excited singlet state. This limits the *f* of singlet formation to 1/4, leading to low UC efficiencies. However, the quintet and triplet (T_2_) states may re-participate in singlet formation *via* other channels like Q_1_ to T_1_ dissociation, and T_2_ to T_1_ internal conversion (IC).^12,13^ This recycling can increase the experimentally obtained *f* value even up to ∼1/2.^5,14,15^ The TTA-UC (^1^(T_1_T_1_) > S_1_S_0_) is the reverse process of singlet fission (S_1_S_0_ > ^1^(T_1_T_1_)) with an intermediate correlated triplet-pair state, ^1^(T_1_T_1_) as per the Johnson–Merrifield model eqn (3).^16,17^

3 SF → S_0_S_1_ ⇌ T_1_T_1_ ⇌ T_1_ + T_1_ ← TTA-UC

It is according to the Merrifield model that the singlet character of the T_1_T_1_ pair determines its coupling to the singlet state.^18^ Hence, annihilators with a triplet-pair state exhibiting a significant singlet character can have a high probability of singlet formation and consequently, a higher *f* factor^19^ which has been investigated in this work by calculating the percentage of charge resonance/transfer character in the S_0_S_1_ dimer.

Another way to increase the *f* factor is to avoid secondary loss channels such as 2T_1_ to T_*n*_ non-radiative decay (Scheme 1b). This can be achieved *via* the molecular engineering of an annihilator with 2T_1_ ≈ S_1_ and Q_*n*_ and T_*n*_ states higher in energy than the 2T_1_ state. This prevents 2T_1_ decay to Q_*n*_ or T_*n*_ states due to the energy gap law relation^5^ (eqn (3)). This results in preferential decay of 2T_1_ to the S_1_ state, leading to more efficient singlet generation.4
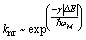
where *k*_nr_ is the rate of non-radiative decay and Δ*E* is the energy gap between electronic states.

Several derivatives of naphthalene,^14,20^ anthracene,^21–23^ perylene,^24,25^ rubrene,^26–30^ and diketopyrrolopyrrole^31,32^ based compounds have been investigated to achieve high *f* values to boost the overall *ϕ*_UC_.^5^ The UC emission of these compounds spans across the majority of the UV-visible spectrum. However, the lack of an efficient annihilator emitting within the 470–540 nm range impedes important biological applications which can be photoactivated with upconverted green light upon excitation within or close to the phototherapeutic window (650–850 nm,^33^ These applications include targeted drug delivery,^34^ light–gated ion channel control,^35^ light-activated CRISPR,^36^ photo-pharmacology,^37^ and photosynthesis.^38^ While 9,10-bis(phenylethynyl)anthracene (BPEA) is a well-known commercially available green annihilator, its low UC quantum yield due to the small *f* = 5.6 to 6.3% is an issue.^21^ Therefore, an efficient annihilator within the 470–540 emission range could serve as a powerful photoactivation tool in biological applications upon low-density red or NIR excitation *via* TTA-UC for embracing higher penetration into biological tissue.

**Fig. 1:**
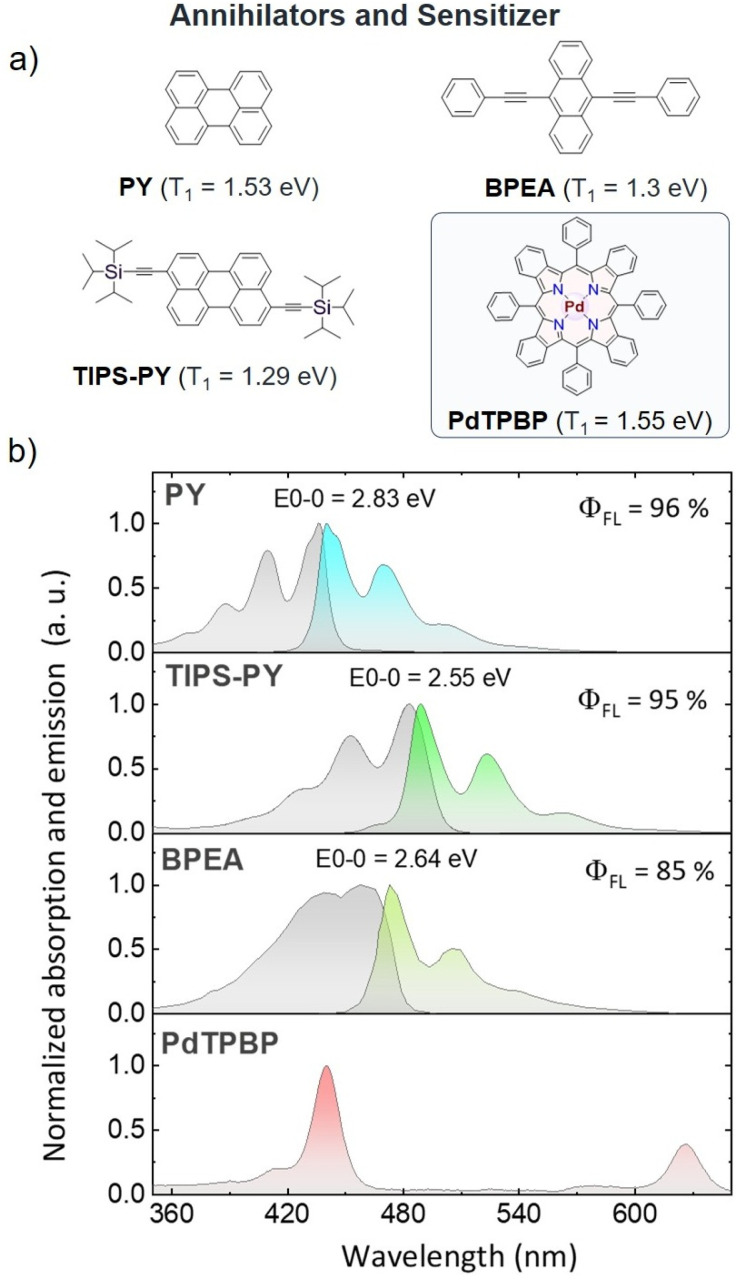
Molecular structures and T_1_ energies (a), and absorption and emission spectra (b) of annihilators (PY, TIPS-PY, BPEA), and sensitizer (PdTPBP) at concentrations of 20 μM and 1 μM in THF, respectively.

Herein, we report the synthesis of a new perylene (PY) based annihilator functionalized with triisopropylsilyl (TIPS) groups, TIPS-PY (Fig. 1a). TIPS-PY demonstrates highly efficient TTA-UC, with experimental *ϕ*_UC_ of 13.7% (out of 50%), with *f* = 39.2% upon combining with Pd(ii) *meso*-tetraphenyl tetrabenzoporphine (PdTPBP) as sensitizer (*λ*_ex_ = 640 nm CW laser). The *ϕ*_UC_ is shown to be greater compared to unfunctionalized PY due to the increase in *f* value implying enhanced triplet-pair-singlet coupling, which may be governed by the singlet-like character of the triplet-pair state of TIPS-PY, revealed from the higher percentage charge resonance or charge transfer character of the S_0_S_1_ excitations of the TIPS-PY compared to PY using TheoDORE program.^19,39^ To our knowledge, an efficient annihilator in 470–540 nm emission range with a high *f* value of 39.2% ± 2.4% and *ϕ*^∞^_UC_ up to 19.6% (theoretical limit) has not been reported before.^40^ This study demonstrates the value of TIPS-functionalization in engineering the triplet energy, singlet-triplet character, and T_1_T_1_ coupling of annihilator triplets to yield a high statistical probability factor for upconverted singlet-state generation, which is a key limiting factor in TTA-UC. When combined with Os(m-peptpy)_2_(TFSI)_2_ as a sensitizer, TIPS-PY upconverted the 730 nm light into yellow-green light, thus reaching deep into the phototherapeutic window^33^ that is highly sought after for various biological applications.^34–38^

## Results and discussion

### Synthesis of 3,9-bis((triisopropylsilyl)ethynyl)perylene (TIPS-PY)

**Scheme 2:**
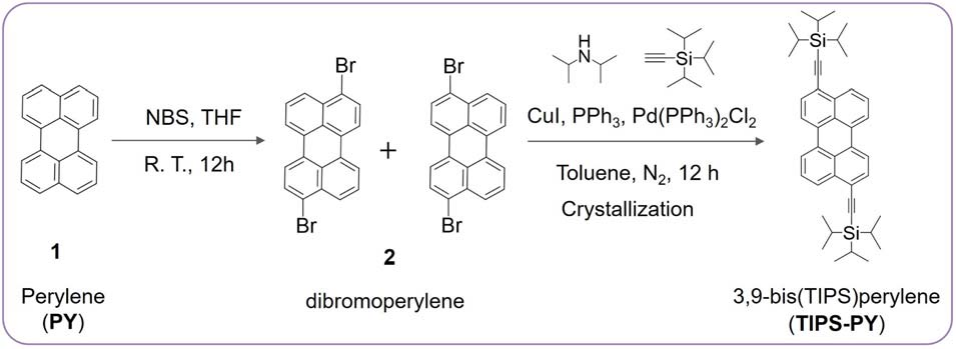
Synthesis of 3,9-bis(TIPS)perylene (TIPS-PY).

The annihilator TIPS-PY was synthesized in a two-step reaction as depicted in Scheme 2. A mixture of 3,9- and 3,10-dibromoperylene was obtained *via* an electrophilic aromatic bromination reaction between PY, and *n*-bromosuccinimide (NBS).^41^ The final compound, TIPS-PY, was obtained *via* a Sonogashira coupling between the mixture of 3,9- and 3,10-dibromoperylene and TIPS-acetylene. The purified-orange colored compound was characterized by ^1^H NMR, ^13^C {^1^H} NMR, MALDI-TOF, and single-crystal X-ray diffraction analysis (for detailed synthesis procedure and characterization see Annexure 1, Fig. S1–S5) and found to be 3,9-bis(TIPS)perylene.

### Photophysical properties

The photophysical properties of TIPS-PY were studied in comparison to other competitive annihilators, PY and BPEA in the 470 to 540 nm emission range. The molecular structures of TIPS-PY, PY,^42^ and BPEA (ref. 21) are shown in Fig. 1a along with PdTPBP, the sensitizer used in this study for red-to-green upconversion.^43^

TIPS-PY demonstrated absorption and emission peaks at 483 nm (*ε* ∼73 000 M^−1^ cm^−1^, Fig. S6) and 489 nm (*ϕ*_FL_ = 95% and *τ*_FL_ = 5.5 ns), respectively (Fig. 1b, S7, and S8). Compared to PY, the emission spectrum of TIPS-PY is red-shifted by 0.29 eV due to the extension of conjugation upon introduction of TIPS-acetylene moieties (Fig. 1b). However, the *Φ*_FL_ remained almost the same (96% and 95%). This red shift in the emission spectrum of TIPS-PY overcame the secondary inner filter issue caused by reabsorption of UC light by the PdTPBP Soret band to boost the *ϕ*_UC_ (Fig. S9). When compared to BPEA, the emission spectrum of TIPS-PY is red-shifted by just 0.1 eV (Fig. 1b). However, the lower *ϕ*_FL_ = 85%^21^ of BPEA implies a negative effect on the overall *ϕ*_UC_ according to eqn (1). Besides *ϕ*_FL_, our previous time-dependent density functional theory (TD-DFT) investigations (Gray *et al.*^21^) found that the difference in geometry of singlet and triplet surfaces of BPEA makes the triplet-state energetically inefficient to generate the first excited singlet-state to yield low *ϕ*_UC_.^21^ Hence, prior to TTA-UC experiments, molecular geometry optimization, and excited state modeling studies of TIPS-PY in comparison to PY were conducted (Fig. 1 and S10).

### Excited-state modeling studies

The DFT and TD-DFT calculations carried out at the (U)PBE0-D3(BJ)/6-311G(d,p) level of theory have shown the T_1_ states of PY and TIPS-PY at 1.49 eV and 1.29 eV, respectively (see SI for a more detailed description of the computational method). The calculated T_1_ of PY is almost similar to the reported experimental^8^ and theoretical values ∼1.5 eV.^42^ The calculated singlet-state (S_1_) energies of PY and TIPS-PY (Fig. S10) are also in agreement with the experimental S_1_ values. Due to the T_1_ of TIPS-PY at 1.29 eV, PdTPBP having T_1_ at 1.55 eV^42^ (Fig. 1a) was selected as a sensitizer to ensure the feasible sensitization of TIPS-PY*via* an exothermic triplet energy transfer pathway. Moreover, the non-overlapping of the emission spectrum of TIPS-PY with the absorption spectrum of PdTPBP (Fig. 1b and S9), and a high *ϕ*_ISC_ of PdTPBP approaching unity^44^ were other key factors for PdTPBP selection as a sensitizer.

**Fig. 2:**
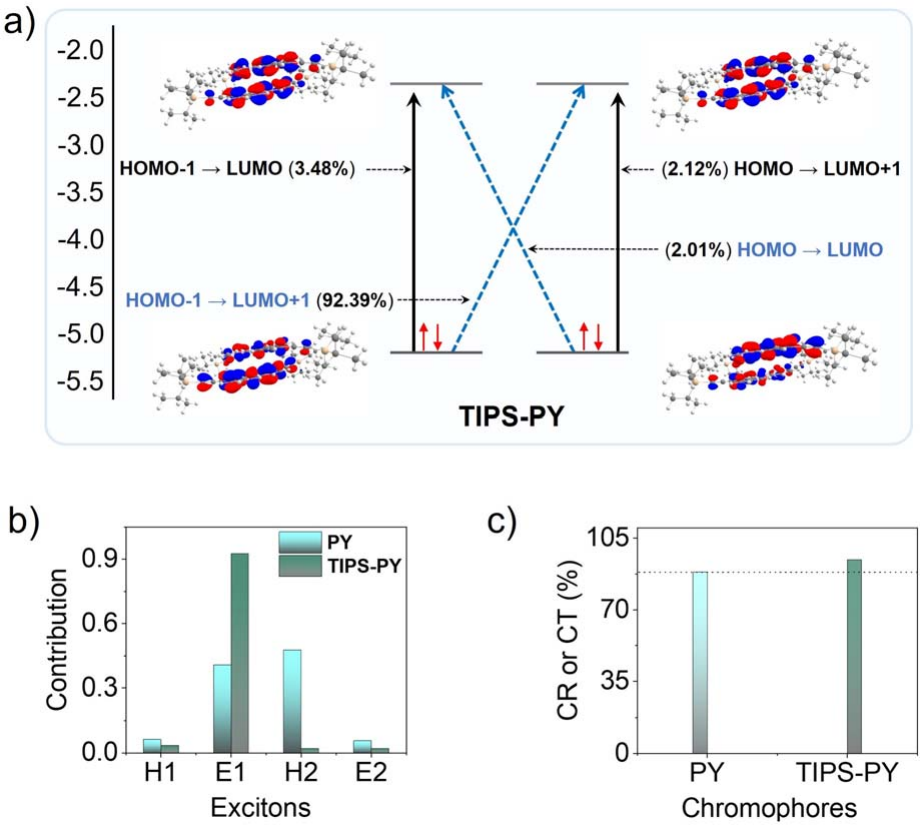
(a) Illustration of the contribution of the linear combination of orbital replacement in TIPS-PY. (b) Plot showing comparative electron (E), and hole (H) contributions during orbital replacement in PY and TIPS-PY. (c) Percentage of charge resonance (CR) or charge transfer (CT) calculated from E and H contributions during orbital replacement in PY and TIPS-PY.

The calculated energy level distributions (Fig. S10) demonstrate that TIPS-PY complies with the 2T_1_ > S_1_ energetic condition for TTA-UC to occur.^5^ However, the proximity of 2T_1_ to higher energy triplet-states (T_*n*_ = T_2_, T_3_) plays a crucial role in the probability of singlet generation due to the energy gap law relation (eqn (4)), imposing a non-radiative decay channel if 2T_1_ is in the vicinity of T_2_ and further from S_1_.^45,46^ We investigated the implication of the energy gap law in affecting the *f* factor of TIPS-PY and found a 2*E*_T1_-*E*_T2_ energy gap of +70 meV. The gap is significant enough to substantially reduce the non-radiative decay.^45–47^ Hence, it could be one of the key contributors to the high *f* factor of TIPS-PY. However, when compared with the 2*E*_T_1__–*E*_T_2__ = −140 meV of PY having *f* value of 17.9%^42^ this parameter does not seem enough to explain the high *f* factor observed of TIPS-PY. Hence, we explored another possible channel to understand the high *f* factor of TIPS-PY. One key argument of the Merrifield model of triplet–triplet coupling is that the efficiency of singlet generation depends on the triplet pair-singlet coupling.^18,29,48^ Hence, a higher singlet character of the triplet-state can increase the singlet-triplet coupling post triplet–triplet annihilation to generate a high singlet population.^18^ Therefore, we calculated the charge resonance or charge transfer character of S_1_S_0_, commonly shared by ^1^(T_1_T_1_), of TIPS-PY compared to that of PY to assess the singlet character using TheoDORE program (Fig. 2).^39^Fig. 2a shows the change in electron density from the ground state to the excited state, resulting from a linear combination of orbital replacement involving charge transfer (blue arrows) and local excitations (black arrows). The HOMO-1 to LUMO+1 transition in TIPS-PY, which contributes the strongest (92.39%), has charge transfer (CT) character, moving one electron each from left to right and from right to left. Contrary to this, the HOMO−1 to LUMO+1 transition in PY, which contributes the strongest (88.24%), has a charge resonance (CR) character. The percentage of CT or CR character is indicative of the singlet character of the dimer.^39^ These results indicate that TIPS-functionalization increases the singlet character of the TIPS-PY dimer, which is likely to have a positive effect on *f* value (Scheme 1b)^16,49–51^ and UC quantum yield.

To substantiate these results, we also investigated biphenyl (BP) and bis-TIPS-biphenyl (TIPS-BP), having a similar transition dipole axis as that of PY (Fig. S11a and b)^52,53^ using the TheoDORE program (Fig. S12). Similar to PY, the CT character of BP increased upon TIPS-functionalization (Fig. S12d), thus supporting the proposed argument. Recently, TIPS-BP was shown to demonstrate superior UC performance compared to BP, confirming our prediction experimentally.^54^ Seeking further generalization of this argument, we also calculated the CT or CR character for highly efficient TIPS-functionalized annihilators such as TIPS-anthracene (TIPS-An) (ref. 23) and TIPS-naphthalene (TIPS-Naph).^55^ However, the CT or CR character decreased for these molecules upon TIPS-functionalization (Fig. S13a–d). This could be due to the difference in the main transition dipole axis of Naph and An (^1^L_*α*_), which unlike BP and PY is along the horizontal axis (Fig. S11b). It is to mention that the transition dipole axis plays a key role in the electronic interactions of the molecules in the excited state.^56^ Nevertheless, it shows that the singlet character of the triplet pair may not be the sole criterion to evaluate the high TTA-UC quantum yields in molecules with different transition dipole axes. Therefore, we also investigated the role of energy gap law^5^ in TIPS-An and TIPS-Naph and found 2*E*_T_1__–*E*_T_2__ of −103 meV, and +186 meV, respectively (Table S2), which is in synergy with results obtained upon application of the energy gap law in the case of PY and TIPS-PY. Therefore, the energy gap law could be the common factor contributing to high *ϕ*_UC_ (27%)^23^ of TIPS-An and high *f* value (54%)^55^ of TIPS-Naph chromophores, as well as TIPS-PY. Seeking further insights, we also computed the S_0_S_0_ and T_1_T_1_ states for PY and TIPS-PY and found a smaller dimerization energy for TIPS-PY (−31.6 kcal mol^−1^) compared to that of PY (−20.43 kcal mol^−1^) (see Fig. S14).^57^ In both cases, the potential energy surface is relatively flat, allowing for easy rotation and translation of the dimers. In the particular case of PY, we have also computed the S_0_S_1_, S_0_T_1_, S_0_T_2_, and T_1_T_2_ states (Fig. S15). As the emission of TIPS-PY is red-shifted due to the presence of TIPS moieties, the extended conjugation leads to a decrease of excited state energies (Fig. 1a) as well as the polarization of the C≡C bond in the opposite direction by the triplet spin compared to PY (Fig. S16). The higher stability of the T_1_ state in TIPS-PY can be attributed to the reduction of the HOMO–LUMO gap by 0.37 eV in TIPS-PY compared to PY.^57^ Given the extension of conjugation, the T_1_ energy of TIPS-PY (1.29 eV) decreased by 0.24 eV compared to PY (1.53 eV) making it suitable for exothermic triplet-energy transfer.

### Triplet–triplet annihilation photon upconversion

**Fig. 3:**
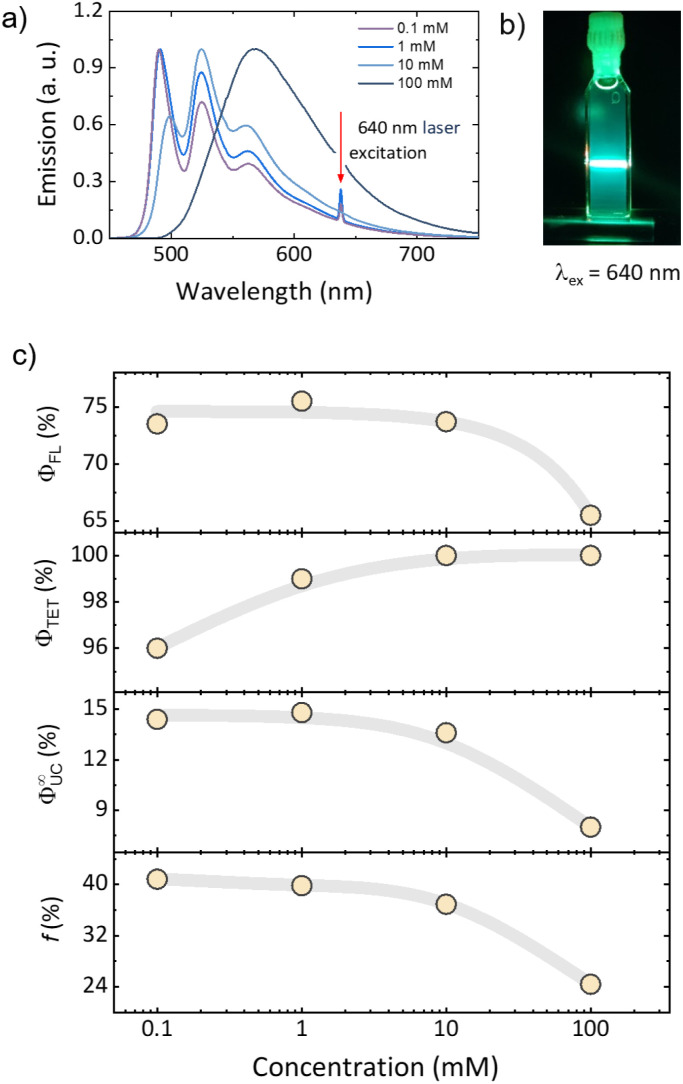
(a) TIPS-PY: PdTPBP upconversion spectra at 0.1 mM, 1 mM, 10 mM, and 100 mM annihilator concentrations. 640 nm laser excitation indicated. (b) Digital image of TTA-UC emission, and (c) *ϕ*_FL_, *ϕ*_TET_, *ϕ*^∞^_UC_, and *f* dependence on TIPS-PY concentration. All solutions were prepared in deaerated THF. PdTPBP concentration in all UC solutions was maintained at 0.01 mM. The grey lines serve as a guide to the eyes.

Following photophysical characterization and excited-state modeling studies, the TIPS-PY annihilator was applied in TTA-UC in combination with PdTPBP as a sensitizer in deaerated THF. The investigated TIPS-PY: PdTPBP UC system demonstrated UC emission upon 640 nm laser excitation (Fig. 3a, b and S17), confirming the DFT prediction of the most favourable energetic condition (2T_1_ ≥ S_1_) for TTA-UC.^5^ To demonstrate the full potential of TIPS-PY, the annihilator concentration was varied from 0.1 mM to 100 mM while the PdTPBP concentration was maintained at 0.01 mM (Fig. 3a and c). The TIPS-PY: PdTPBP system demonstrated a high experimental *ϕ*_UC_ varying from 7.0% to 13.7% (Fig. 3c and Table 1) at 100 mM and 1 mM annihilator concentrations, respectively. The *ϕ*^∞^_UC_ and UC threshold (*I*_th_) were estimated from *ϕ*_UC_*vs.* excitation power density (*I*_ex_) profile according to previously reported procedures^58^ (Fig. S18 and Table 1).

**Table 1:** UC parameters of TIPS-PY-PdTPBP UC solutions in deaerated THF at 0.1 mM, 1 mM, 10 m M, 100 mM, and 0.01 mM concentrations of TIPS-PY and PdTPBP

TIPS-PY	*ϕ*_FL_ a, %	*ϕ*_UC_ b, %	*ϕ* ^∞^_UC_ c, %	*ϕ*_TET_ d, %	*I*_th_ e, W cm^−2^	*τ*_*T*_ f, *μs*	*f* g, %
0.1 mM	73.5	11.1	14.4	96	0.19	1250	40.8
1 mM	75.5	13.7	14.9	99	0.29	914	39.8
10 mM	73.7	13.0	13.6	100	0.43	741	36.9
100 mM	65.5	7.0	8.0	100	4.94	30–80	24.4

a FL quantum yield of annihilator in UC solution.

b Reabsorption corrected maximum measured UC quantum yield values.

c Maximum attainable UC quantum yield values.

d TET quantum yield.

e UC threshold at 38.2% of *ϕ*^∞^_UC_.

f Triplet lifetime (=2 × *τ*_UC_).

g Statistical probability of singlet generation from two triplets *via* TTA, calculated according to eqn (1). *τ*_UC_ values in Table 1 were determined from the tail fit of the UC emission decay profiles in Fig. S21.

The *I*_th_ values for TIPS-PY: PdTPBP vary from 0.19 W cm^−2^ to 0.43 W cm^−2^, a low threshold barrier desired for most applications. The difference in *ϕ*^∞^_UC_ at varying TIPS-PY concentrations can be explained by concentration effects on *ϕ*_FL_ and *ϕ*_TET_ according to eqn (1). While the *ϕ*_FL_ (75.5–73.5%) for 0.1 mM to 10 mM concentrations are similar, the 100 mM concentration sample demonstrates a decrease in *ϕ*_FL_ to 65.5% owing to the aggregation of the annihilator species (Fig. 3a and S19). This suggests an enhanced non-radiative decay channel, potentially due to the aggregation. It was also reflected in the anti-Stokes shifts, which varied from 0.56 to 0.21 eV between 0.1 to 100 mM TIPS-PY (Table S3).^59^ No significant aggregation is observed up to a concentration of 10 mM, as evidenced by the absence of changes in the low-energy shoulder of the absorption spectra (Fig. S20). The growth of *ϕ*_TET_ from 96% to 100% is explained by the higher concentration of acceptor chromophores surrounding sensitizer molecules. The longest triplet-lifetime, *τ*_T_ = 1250 μs was observed at the lowest TIPS-PY concentration (0.1 mM), and decreased further upon increasing the concentration (Table 1 and Fig. S21).

This decreases the average distance between PdTPBP and TIPS-PY, inferring higher TET probability. *ϕ*_TET_ was evaluated *via* rise time (*τ*_r_) of TTA-UC transients (Fig. S22 and Table S4) according to the following relation:5
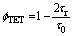
where *τ*_0_ – intrinsic (unquenched) triplet lifetime of the sensitizer that, in the case of PdTPBP, is 175.5 μs.^42^ A high *ϕ*_TET_ = 96% was also confirmed from the quenching of the phosphorescence spectrum of PdTPBP by TIPS-PY (Fig. S22).

To further understand the higher TTA-UC quantum yields obtained with TIPS-PY, the *f* value of 39.2% ± 2.4% was evaluated according to eqn (1) as the average of 3 measurements at 0.1 mM, 1 mM, and 10 mM annihilator concentrations (Fig. 3b and Table 1). The results obtained at 0.1 mM, 1 mM, and 10 mM support that *f* value is an intrinsic property of a molecule that does not experience a change due to the change in concentration. The *f* value of 24.4% recorded at 100 mM was omitted from the calculation due to the presence of TIPS-PY aggregates (Fig. 3a and S19) in the UC solution enabling non-radiative decay channels.

**Fig. 4:**
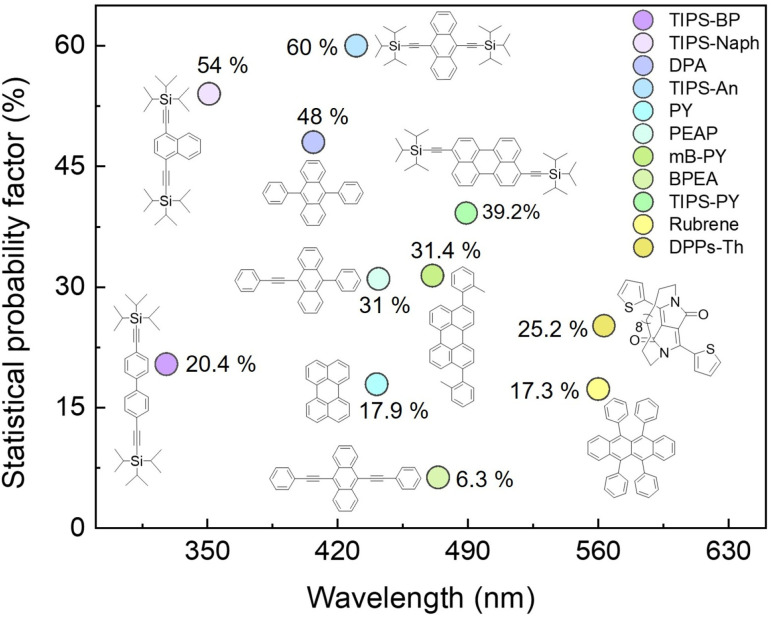
Plot showing variation in statistical probability factor of various annihilators emitting across the visible spectral range. TIPS-PY synthesized in this work tops the list in the 470–570 nm range.

For a reliable comparison of *f* values between studied PY,^42^TIPS-PY, and BPEA, we conducted additional measurements at identical conditions with BPEA:PdTPBP UC system to determine the *f* value resulting in 6.3% (Fig. S23, S24, and Table S6). The reported *f* value of 39.2% ± 2.4% for TIPS-PY outperforms all previously studied annihilators within the 470–570 nm emission region and is among the top values in the entire spectrum (Fig. 4).^5,40,54,60–62^ This leads to a high experimental *ϕ*_UC_ of 13.7% with a possibility to approach the intrinsic limit *ϕ*^∞^_UC_ ∼19.6% if all energy transfer processes approach unity. The main reason for the high *f* value of TIPS-PY is the TIPS functional groups, which increase the stability of the triplet state as well as form a singlet-like character of the triplet dimer (T_1_T_1_) species, as revealed from the charge resonance or charge transfer studies. This may exhibit a positive impact on T_1_⋯T_1_ pair state and S_1_ coupling to generate the singlet-state with high efficiency according to the Merrifield model.^18,29,48^ Additionally, the favourable energy distribution prevents 2T_1_-to-T_2_ non-radiative decay from favouring the S_1_ formation.

**Fig. 5:**
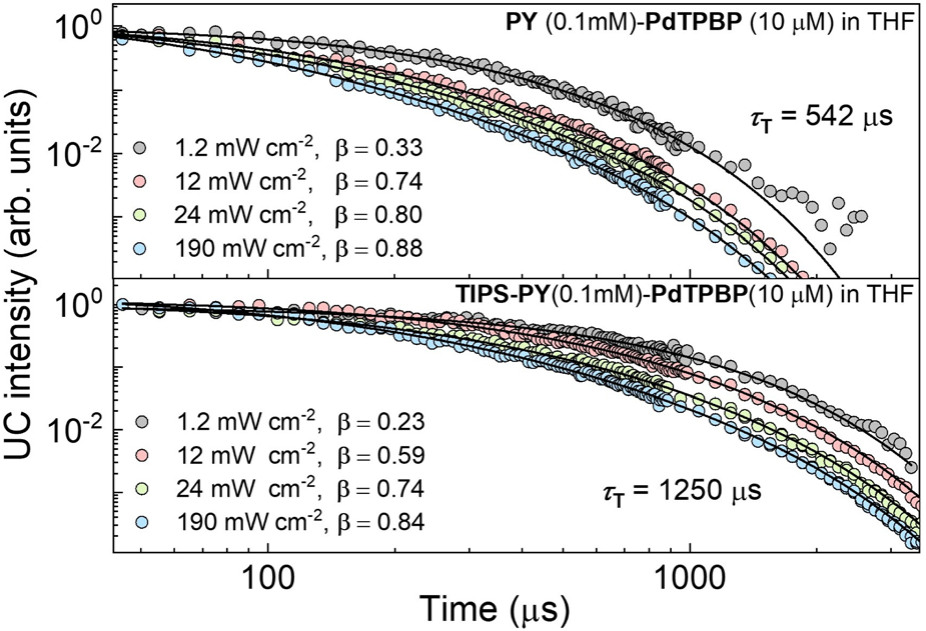
Normalized UC transients of PY and TIPS-PY on a log–log scale at different excitation densities (indicated) upon sensitization with PdTPBP at 640 nm. Circles present experimental data, while solid lines show global fits with a shared *τ*_T_.

We also investigated the rate of TTA (*k*_TTA_) as a possible reason for higher UC performance in TIPS-PY compared to PY in THF. To determine *k*_TTA_ of TIPS-PY, UC intensity decay profiles of TIPS-PY: PdTPBP and PY: PdTPBP solutions containing 0.1 mM of annihilator were measured at increasing excitation power densities (Fig. 5) and fitted using the following relation.^63^6
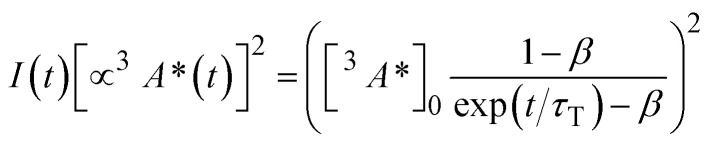
7
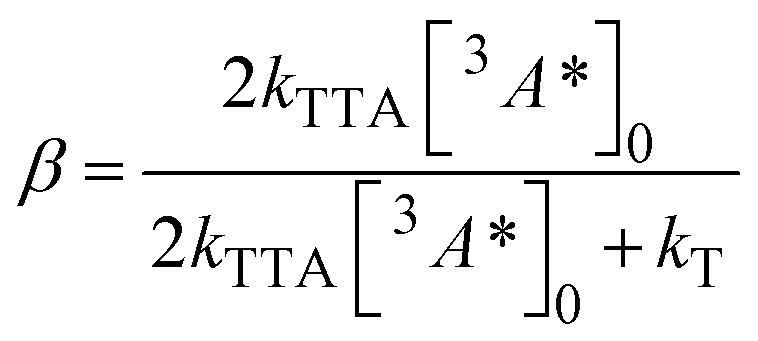
Here, [^3^A*]_0_ denotes the initial triplet exciton concentration within the annihilator, and *τ*_T_ (=1/*k*_T_) is the spontaneous triplet decay lifetime. *τ*_T_ was obtained from the tails of the transients, assuming that the condition *k*_T_ >> *k*_TTA_[^3^A*]_0_ is met at low triplet exciton concentration, where TTA is negligible.

The obtained *β* values, which describe TTA efficiency at particular excitation densities, are listed in Table S5, along with *τ*_T_ values. Since pulsed nanosecond excitation with a pulse duration much shorter than triplet lifetime was used, quasi-steady-state conditions could not be achieved, preventing direct determination of [^3^A*]_0_ from these measurements. However, given that the *τ*_T_ is known and remains invariant with excitation density, [^3^A*]_0_ at each pump level was estimated utilizing the reported *k*_TTA_ value for PY (19 × 10^8^ M^−1^ s^−1^ (ref. 64) according to the eqn (7). Considering the similar TET efficiencies for PY: PdTPBP (*ϕ*_TET_ = 92%)^42^ and TIPS-PY: PdTPBP (*ϕ*_TET_ = 96%; this work) at equivalent annihilator and sensitizer concentrations, the [^3^A*]_0_ values derived for PY: PdTPBP were also employed to estimate *k*_TTA_ in TIPS-PY. The estimated *k*_TTA_ for TIPS-PY is approximately 5 × 10^8^ M^−1^ s^−1^, which is 4-fold lower than that of PY. Hence, *k*_TTA_ may not be the reason for higher UC performance in TIPS-PY compared to PY. This observation is different from what has been reported by Han *et al.*^40^ where the higher normalized triplet–triplet annihilation efficiency of 3,10-di-*o*-tolylperylene (mB-PY) triplets, due to the restricted motion of *o*-tolyl rings was cited as the key reason for enhanced UC quantum yield.^40^ Nevertheless, the enhancement of UC performance by TIPS groups provides a novel strategy for molecular design for future annihilators as well as demonstrates the potential of TIPS-PY as another suitable compound to be implemented in numerous applications, especially in biology requiring 470–540 nm emission upon excitation with deep tissue penetrative red/far-red light.

**Fig. 6:**
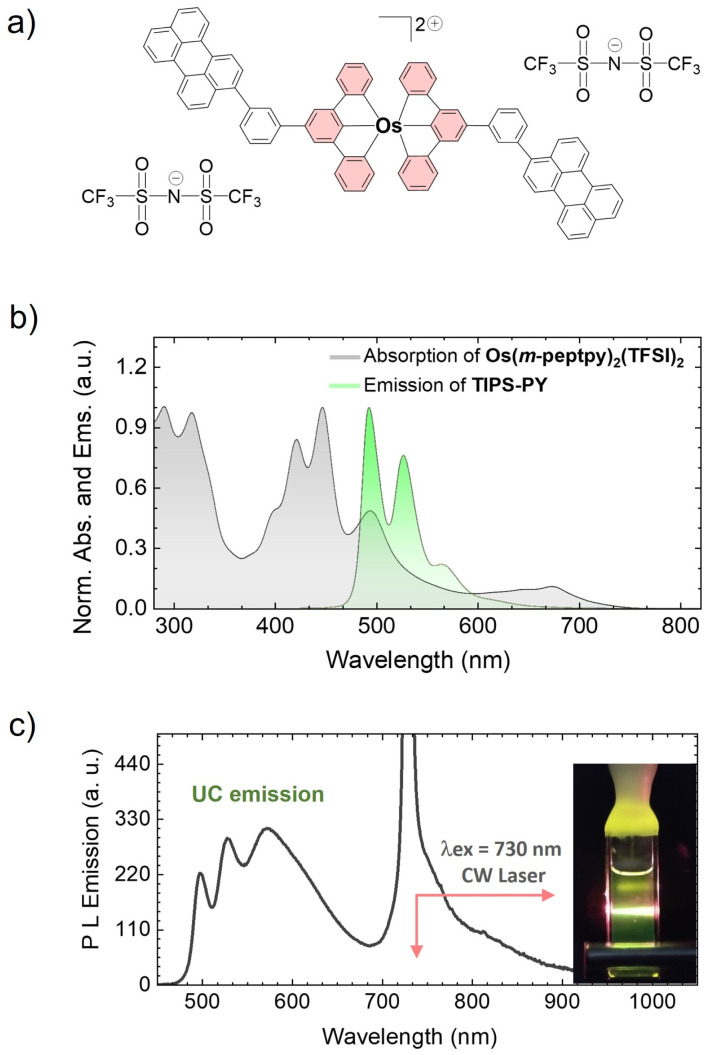
(a) Molecular structure of Os(m-peptpy)_2_(TFSI)_2_. (b) Absorption spectrum of Os(m-peptpy)_2_(TFSI)_2_ and fluorescence emission spectrum of TIPS-PY (1 mM) in the presence of Os(m-peptpy)_2_(TFSI)_2_ (0.01 mM) in DMF (*λ*_e*x*_ = 420 nm). (c) Upconversion emission spectrum of TIPS-PY: Os(*m*-peptpy)_2_(TFSI)_2_ (1 mM:0.01 mM) system (*λ*_ex_ = 730 nm CW laser). (d) Digital image of the yellow-green UC emission upon 730 nm CW laser excitation.

To further demonstrate the potential of the TIPS-PY for long-wavelength far-red light upconversion, we performed TTA-UC study by combining TIPS-PY with Os(*m*-peptpy)_2_(TFSI)_2_ as sensitizer in deaerated DMF (Fig. 6a). The absorption spectrum of Os(*m*-peptpy)_2_(TFSI)_2_ shows vibronic peaks at 291, 318, 422, 447 nm due to ligand centred, at 494 nm due to singlet metal-to-ligand charge transfer (^1^MLCT), and at 648 nm and 673 nm due to triplet MLCT (^3^MLCT) (Fig. 6b).^65^ Furthermore, Os(*m*-peptpy)_2_(TFSI)_2_ shows ^3^MLCT emission at 759 nm (1.63 eV) and phosphorescence emission due to meta-substituted perylene units at 827 nm (1.5 eV), respectively.^65^ Unlike other Os-complexes, it shows a long phosphorescence lifetime (*τ*_Po_) of 98 μs (Fig. S25), which is among the key requirements for an efficient sensitizer in TTA-UC.^5^ Upon excitation with a 730 nm CW laser, the TIPS-PY:Os(*m*-peptpy)_2_(TFSI)_2_ (1 mM:0.01 mM) system demonstrated bright yellow-green UC emission (Fig. 6c and d), thus expanding the upconversion range into the phototherapeutic window to the far-red region.^33^ The phosphorescence transients (Fig. S25) were used to determine the *ϕ*_TET_ = 99.7%, indicating almost complete quenching of TIPS-PY:Os(*m*-peptpy)_2_(TFSI)_2_ phosphorescence by TIPS-PY. Despite the high *ϕ*_TET_, long triplet lifetime, *τ*_T_ = 846 μs (Fig. S26) and high *ϕ*_FL_ = 71% of TIPS-PY in this system, a low absolute *ϕ*_UC_ = 0.62% was observed. This could be due to 1) the secondary inner filter effect caused by fast reabsorption of the upconverted light by Os(*m*-peptpy)_2_(TFSI)_2_ due to the high spectral overlap of its absorption spectrum with the emission spectrum of TIPS-PY (Fig. 6b and Fig. S27), or 2) aggregated UC emission due to complexation of TIPS-PY with Os(*m*-peptpy)_2_(TFSI)_2_ confirmed from the distorted UC emission spectrum showing shift in emission maxima to 572 nm in the yellow emission range (Fig. 6c). Comparatively low solubility of TIPS-PY in DMF also support the possible aggregated UC emission. A better far-red absorbing sensitizer with higher transparency window in the TIPS-PY emission range may yield higher *ϕ*_UC_.

## Conclusions

We synthesized a new green-emitting annihilator TIPS-PY, which exhibits the highest absolute TTA-UC quantum yield of 13.7% (50% theoretical maximum) for red-to-green (640 nm to 489 nm) TTA-UC upon combining with PdTPBP as a sensitizer. Such a high UC quantum yield is enabled by the combined effects of: (1) a high *ϕ*_FL_ = 95% of TIPS-PY, (2) *ϕ*_TET_ ∼100% due to exothermic triplet energy transfer from PdTPBP to TIPS-PY, (3) minuscule secondary inner filter effects due to minimum spectral overlap of PdTPBP absorption and TIPS-PY emission, and (4) a high *f* value of 39.2% ± 2.4% of TIPS-PY, which generated a high singlet population after triplet-coupling. Further investigations of the *f* factor from the TheoDORE program revealed a singlet-like character of the triplet-pair state of TIPS-PY induced by the higher charge transfer character of the S_0_S_1_ excitations of TIPS-PY compared to PY. This may increase the coupling of the triplet-pair state with the excited singlet state of TIPS-PY to generate a high singlet population after triplet–triplet annihilation according to the Merrifield model. Interestingly, this behaviour was also observed for other TIPS-functionalized annihilators like TIPS-BP, having a similar transition dipole axis to that of PY, which was not previously explored. The obtained *ϕ*_UC_ and *f* values for TIPS-PY are among the highest for annihilators in the green-to-red spectral range and outperform well-known annihilators such as PY, BPEA, mB-PY, rubrene, or DPPs.

## Author contributions

P. B., L. N., and K. M. P. conceptualized the idea of this work. M.M. synthesized the TIPS-Perylene. P. B. and M. M. carried out primary photophysical characterization. L. N., M. D., E. R, G. K., P. B., and K. K. carried out UC and time resolved measurements. C. A. and M. S. carried out computational calculations. Y. S., N. Y., and N. K. synthesized Os-complex. P.B., L. N., and K. M. P. wrote the first draft of the manuscript. All authors contributed to the analysis of results in their respective parts and editing of the manuscript.

## Conflicts of interest

There are no conflicts to declare.

## Supplementary Material

SC-016-D5SC05248C-s001

SC-016-D5SC05248C-s002

## Data Availability

The original datasets generated and analyzed during the current study are available from the corresponding authors on reasonable request and also available via open access on Zenodo at https://doi.org/10.5281/zenodo.3755810. Supplementary information: synthesis, NMR, MALDI-TOF, and X-Ray diffraction analysis of TIPS-PY together with photophysical and upconversion measurements and DFT calculations. The authors have cited additional references within the SI.^66–84^ Cartesian coordinates of the species studied for computational calculations. See DOI: https://doi.org/10.1039/d5sc05248c.
