# Supplementary material for: Enhancing the statistical probability factor in triplet–triplet annihilation photon upconversion via TIPS functionalization
Source: Chem Sci. 2025 Oct 7;16(43):20255–64. doi: 10.1039/d5sc05248c (PMC12519238; doi:10.1039/d5sc05248c)
Supplement: SC-016-D5SC05248C-s001 [file SC-016-D5SC05248C-s001.pdf]

Supplementary Information (SI) for Chemical Science.
This journal is © The Royal Society of Chemistry 2025

Supporting Information-2

Cartesian coordinates of the species studied in

Enhancing the Statistical Probability Factor in Triplet-Triplet Annihilation Upconversion via TIPS Functionalization

Lukas Naimovičius,[a,b,c] Manvydas Dapkevičius,[b] Edvinas Radiunas [b] Mila Miroshnichenko,[a] Gediminas Kreiza [b] Carles Alcaide,[d] Paulius Baronas,[e] Yoichi Sasaki,[f] Nobuhiro Yanai,[f] Nobuo Kimizuka,[f] Andrew B. Pun,[c] Miquel Solà,[d] Pankaj Bharmoria,*[a] Karolis Kazlauskas,[b] and Kasper Moth-Poulsen*[a,e,g,h]

[a] Institute of Materials Science of Barcelona, ICMAB-CSIC, Bellaterra, Barcelona, 08193, Spain.
[b] Institute of Photonics and Nanotechnology, Vilnius University, Saulėtekio av. 3, LT-10257 Vilnius, Lithuania.
[c] Department of Chemistry and Biochemistry, University of California San Diego, 92093 La Jolla, CA, USA.
[d] Institute of Computational Chemistry and Catalysis (IQCC), Universitat de Girona, M. Aurèlia Capmany 69, 17003 Girona, Spain.
[e] Department of Chemical Engineering, Universitat Politècnica de Catalunya, EEBE, Eduard Maristany 10–14, 08019 Barcelona, Spain.
[f] Department of Applied Chemistry, Graduate School of Engineering, Kyushu University, 744 Moto-oka, Nishi-ku, Fukuoka 819-0395, Japan.
[g] Catalan Institution for Research & Advanced Studies, ICREA, Pg. Lluís Companys 23, Barcelona, Spain.
[h] Department of Chemistry and Chemical Engineering, Chalmers University of Technology, Kemivagen 4, Gothenburg 412 96, Sweden.

*E-mails – kasper.moth-poulsen@upc.edu; pbharmoria@icmab.es

Table C1. PBE0-D3(BJ)/6-311G(d,p) optimized xyz Cartesian coordinates (Å) for the singlet ground state (S_0) of **PY**.

6	0.000000000	1.227021000	3.557128000
6	0.000000000	0.000000000	2.858816000
6	0.000000000	0.000000000	1.432122000
6	0.000000000	1.242799000	0.734329000
6	0.000000000	2.415632000	1.469560000
6	0.000000000	2.411616000	2.870275000
6	0.000000000	-1.242799000	0.734329000
6	0.000000000	1.242799000	-0.734329000
6	0.000000000	0.000000000	-1.432122000
6	0.000000000	-1.242799000	-0.734329000
6	0.000000000	0.000000000	-2.858816000
6	0.000000000	1.227021000	-3.557128000

6	0.000000000	2.411616000	-2.870275000
6	0.000000000	2.415632000	-1.469560000
1	0.000000000	1.211370000	4.642253000
1	0.000000000	3.355385000	3.404945000
1	0.000000000	1.211370000	-4.642253000
1	0.000000000	3.355385000	-3.404945000
6	0.000000000	-1.227021000	3.557128000
6	0.000000000	-2.411616000	2.870275000
6	0.000000000	-2.415632000	1.469560000
1	0.000000000	-1.211370000	4.642253000
1	0.000000000	-3.355385000	3.404945000
6	0.000000000	-2.415632000	-1.469560000
6	0.000000000	-1.227021000	-3.557128000
1	0.000000000	-1.211370000	-4.642253000
6	0.000000000	-2.411616000	-2.870275000
1	0.000000000	-3.355385000	-3.404945000
1	0.000000000	-3.373424000	0.964807000
1	0.000000000	-3.373424000	-0.964807000
1	0.000000000	3.373424000	-0.964807000
1	0.000000000	3.373424000	0.964807000

Table C2. PBE0-D3(BJ)/6-311G(d,p) optimized xyz Cartesian coordinates (Å) for the first triplet excited state of **PY**.

6	0.000000000	1.224880000	3.536188000
6	0.000000000	0.000000000	2.843809000
6	0.000000000	0.000000000	1.410273000
6	0.000000000	1.236956000	0.710186000
6	0.000000000	2.450645000	1.468320000
6	0.000000000	2.434452000	2.841273000
6	0.000000000	-1.236956000	0.710186000
6	0.000000000	1.236956000	-0.710186000
6	0.000000000	0.000000000	-1.410273000
6	0.000000000	-1.236956000	-0.710186000
6	0.000000000	0.000000000	-2.843809000
6	0.000000000	1.224880000	-3.536188000
6	0.000000000	2.434452000	-2.841273000
6	0.000000000	2.450645000	-1.468320000
1	0.000000000	1.213319000	4.621014000
1	0.000000000	3.370405000	3.389780000
1	0.000000000	1.213319000	-4.621014000
1	0.000000000	3.370405000	-3.389780000
6	0.000000000	-1.224880000	3.536188000
6	0.000000000	-2.434452000	2.841273000
6	0.000000000	-2.450645000	1.468320000

1	0.000000000	-1.213319000	4.621014000
1	0.000000000	-3.370405000	3.389780000
6	0.000000000	-2.450645000	-1.468320000
6	0.000000000	-1.224880000	-3.536188000
1	0.000000000	-1.213319000	-4.621014000
6	0.000000000	-2.434452000	-2.841273000
1	0.000000000	-3.370405000	-3.389780000
1	0.000000000	-3.403865000	0.957982000
1	0.000000000	-3.403865000	-0.957982000
1	0.000000000	3.403865000	-0.957982000
1	0.000000000	3.403865000	0.957982000

Table C3. PBE0-D3(BJ)/6-311G(d,p) optimized xyz Cartesian coordinates (Å) for the first singlet excited state (S_1) of **PY**.

6	0.000000000	1.227570000	3.538365000
6	0.000000000	0.000000000	2.846149000
6	0.000000000	0.000000000	1.417708000
6	0.000000000	1.238206000	0.717487000
6	0.000000000	2.444500000	1.463768000
6	0.000000000	2.430420000	2.845787000
6	0.000000000	-1.238206000	0.717487000
6	0.000000000	1.238206000	-0.717487000
6	0.000000000	0.000000000	-1.417708000
6	0.000000000	-1.238206000	-0.717487000
6	0.000000000	0.000000000	-2.846149000
6	0.000000000	1.227570000	-3.538365000
6	0.000000000	2.430420000	-2.845787000
6	0.000000000	2.444500000	-1.463768000
1	0.000000000	1.216384000	4.623462000
1	0.000000000	3.368076000	3.391346000
1	0.000000000	1.216384000	-4.623462000
1	0.000000000	3.368076000	-3.391346000
6	0.000000000	-1.227570000	3.538365000
6	0.000000000	-2.430420000	2.845787000
6	0.000000000	-2.444500000	1.463768000
1	0.000000000	-1.216384000	4.623462000
1	0.000000000	-3.368076000	3.391346000
6	0.000000000	-2.444500000	-1.463768000
6	0.000000000	-1.227570000	-3.538365000
1	0.000000000	-1.216384000	-4.623462000
6	0.000000000	-2.430420000	-2.845787000
1	0.000000000	-3.368076000	-3.391346000
1	0.000000000	-3.397490000	0.951848000
1	0.000000000	-3.397490000	-0.951848000

1	0.000000000	3.397490000	-0.951848000
1	0.000000000	3.397490000	0.951848000

Table C4. PBE0-D3(BJ)/6-311G(d,p) optimized xyz cartesian coordinates (Å) for the second triplet excited state (T_2) of **PY**.

6	0.000000000	1.237891000	3.546178000
6	0.000000000	0.000000000	2.828403000
6	0.000000000	0.000000000	1.429742000
6	0.000000000	1.245936000	0.710084000
6	0.000000000	2.446440000	1.469539000
6	0.000000000	2.429868000	2.863826000
6	0.000000000	-1.245936000	0.710084000
6	0.000000000	1.245936000	-0.710084000
6	0.000000000	0.000000000	-1.429742000
6	0.000000000	-1.245936000	-0.710084000
6	0.000000000	0.000000000	-2.828403000
6	0.000000000	1.237891000	-3.546178000
6	0.000000000	2.429868000	-2.863826000
6	0.000000000	2.446440000	-1.469539000
1	0.000000000	1.214052000	4.630549000
1	0.000000000	3.369325000	3.406355000
1	0.000000000	1.214052000	-4.630549000
1	0.000000000	3.369325000	-3.406355000
6	0.000000000	-1.237891000	3.546178000
6	0.000000000	-2.429868000	2.863826000
6	0.000000000	-2.446440000	1.469539000
1	0.000000000	-1.214052000	4.630549000
1	0.000000000	-3.369325000	3.406355000
6	0.000000000	-2.446440000	-1.469539000
6	0.000000000	-1.237891000	-3.546178000
1	0.000000000	-1.214052000	-4.630549000
6	0.000000000	-2.429868000	-2.863826000
1	0.000000000	-3.369325000	-3.406355000
1	0.000000000	-3.403328000	0.965492000
1	0.000000000	-3.403328000	-0.965492000
1	0.000000000	3.403328000	-0.965492000
1	0.000000000	3.403328000	0.965492000

Table C5. PBE0-D3(BJ)/6-311G(d,p) optimized xyz Cartesian coordinates (Å) for the singlet ground state (S_0) of **TIPS-PY**.

6	3.697951000	0.769441000	-0.064024000
6	2.836072000	-0.368964000	-0.006909000

6	1.423383000	-0.181792000	-0.011209000
6	0.882875000	1.135872000	-0.037343000
6	1.759379000	2.207734000	-0.105647000
6	3.141360000	2.033384000	-0.121130000
6	0.569592000	-1.323574000	0.012402000
6	-0.569609000	1.323409000	0.012377000
6	-1.423397000	0.181629000	-0.011216000
6	-0.882892000	-1.136037000	-0.037360000
6	-2.836084000	0.368806000	-0.006938000
6	-3.367181000	1.672882000	0.053731000
6	-2.530337000	2.757351000	0.104175000
6	-1.143292000	2.582252000	0.081085000
1	3.794401000	2.896735000	-0.174294000
1	-2.940706000	3.759799000	0.159503000
6	3.367167000	-1.673040000	0.053803000
6	2.530320000	-2.757506000	0.104289000
6	1.143274000	-2.582412000	0.081184000
1	4.444059000	-1.795502000	0.063537000
1	2.940691000	-3.759950000	0.159676000
6	-1.759404000	-2.207894000	-0.105734000
6	-3.697964000	-0.769582000	-0.064063000
6	-3.141387000	-2.033531000	-0.121215000
1	-3.794435000	-2.896874000	-0.174418000
6	5.103780000	0.597416000	-0.065402000
6	6.306940000	0.417640000	-0.062646000
14	8.110666000	0.058547000	-0.007821000
6	8.995985000	1.736616000	-0.387871000
6	8.432298000	-0.589855000	1.788390000
6	8.404106000	-1.289704000	-1.369574000
6	8.371172000	-0.664846000	-2.769192000
1	9.201815000	0.021882000	-2.945636000
1	8.444635000	-1.463033000	-3.519388000
1	7.434848000	-0.129135000	-2.951500000
6	7.277044000	-2.331498000	-1.340185000
1	7.448756000	-3.068204000	-2.136047000
1	7.218913000	-2.877448000	-0.398084000
1	6.304700000	-1.866194000	-1.523244000
6	9.750162000	-1.998202000	-1.184965000
1	9.899654000	-2.720810000	-1.997880000
1	10.593343000	-1.302089000	-1.208513000
1	9.800957000	-2.553613000	-0.245339000
6	7.881863000	-2.011022000	1.957828000
1	8.413762000	-2.747779000	1.352023000
1	7.991104000	-2.314100000	3.007224000
1	6.816483000	-2.063753000	1.712741000

6	9.926182000	-0.603128000	2.130181000
1	10.069192000	-1.019659000	3.135878000
1	10.508151000	-1.216728000	1.436831000
1	10.358813000	0.400587000	2.133730000
6	10.471992000	1.534566000	-0.747860000
1	10.944107000	2.510108000	-0.922994000
1	11.031779000	1.040096000	0.051100000
1	10.599055000	0.945778000	-1.659667000
6	7.690482000	0.280569000	2.812777000
1	7.842891000	-0.140463000	3.815326000
1	8.043810000	1.311398000	2.834853000
1	6.615098000	0.297534000	2.616717000
6	8.917315000	2.677381000	0.820178000
1	9.491267000	2.313080000	1.675100000
1	9.334342000	3.653879000	0.541243000
1	7.884491000	2.840674000	1.142313000
6	8.289754000	2.457476000	-1.544891000
1	8.771302000	3.430798000	-1.707230000
1	8.338560000	1.908159000	-2.485046000
1	7.235657000	2.638745000	-1.317301000
1	0.516486000	-3.464055000	0.123909000
1	-1.378729000	-3.219987000	-0.152735000
1	-0.516506000	3.463898000	0.123759000
1	1.378698000	3.219827000	-0.152583000
1	-4.444078000	1.795340000	0.063447000
6	-5.103781000	-0.597544000	-0.065440000
6	-6.306947000	-0.417728000	-0.062680000
14	-8.110640000	-0.058496000	-0.007817000
6	-8.432226000	0.589760000	1.788463000
6	-8.404030000	1.289939000	-1.369390000
6	-8.996109000	-1.736454000	-0.388056000
6	-8.917459000	-2.677454000	0.819816000
1	-9.491355000	-2.313305000	1.674843000
1	-9.334560000	-3.653871000	0.540702000
1	-7.884633000	-2.840888000	1.141881000
6	-8.289970000	-2.457113000	-1.545248000
1	-8.771629000	-3.430339000	-1.707835000
1	-8.338709000	-1.907536000	-2.485257000
1	-7.235893000	-2.638558000	-1.317706000
6	-10.472112000	-1.534203000	-0.747957000
1	-10.944338000	-2.509666000	-0.923215000
1	-11.031813000	-1.039784000	0.051090000
1	-10.599135000	-0.945270000	-1.659672000
6	-8.371325000	0.665292000	-2.769116000
1	-9.202068000	-0.021303000	-2.945609000

1	-8.444770000	1.463613000	-3.519176000
1	-7.435091000	0.129490000	-2.951624000
6	-9.749968000	1.998600000	-1.184567000
1	-9.899420000	2.721335000	-1.997376000
1	-10.593239000	1.302597000	-1.208163000
1	-9.800643000	2.553888000	-0.244864000
6	-9.926114000	0.603083000	2.130230000
1	-10.069120000	1.019484000	3.135982000
1	-10.508039000	1.216809000	1.436954000
1	-10.358794000	-0.400608000	2.133639000
6	-7.276781000	2.331519000	-1.339927000
1	-7.448462000	3.068463000	-2.135577000
1	-7.218397000	2.877201000	-0.397692000
1	-6.304554000	1.866065000	-1.523230000
6	-7.881680000	2.010848000	1.958140000
1	-8.413483000	2.747759000	1.352442000
1	-7.990929000	2.313758000	3.007584000
1	-6.816285000	2.063522000	1.713108000
6	-7.690484000	-0.280874000	2.812721000
1	-7.842988000	0.139922000	3.815356000
1	-8.043779000	-1.311719000	2.834512000
1	-6.615085000	-0.297764000	2.616740000

Table C6. PBE0-D3(BJ)/6-311G(d,p) optimized xyz Cartesian coordinates (Å) for the first triplet excited state (T_1) of **TIPS-PY**.

6	3.699830000	0.769450000	-0.034452000
6	2.824347000	-0.360676000	-0.020706000
6	1.407435000	-0.153123000	-0.017878000
6	0.881271000	1.164874000	-0.021470000
6	1.799702000	2.262812000	-0.033134000
6	3.150424000	2.069171000	-0.040315000
6	0.537557000	-1.280851000	-0.010984000
6	-0.525538000	1.364906000	-0.013244000
6	-1.395428000	0.237138000	-0.010337000
6	-0.869070000	-1.080803000	-0.011326000
6	-2.812566000	0.444317000	-0.005701000
6	-3.320896000	1.748916000	-0.001677000
6	-2.461229000	2.847972000	-0.001467000
6	-1.100944000	2.670149000	-0.007536000
1	3.825643000	2.917000000	-0.050164000
1	-2.874075000	3.850971000	0.003427000
6	3.332528000	-1.665398000	-0.010092000
6	2.472700000	-2.764290000	-0.001751000
6	1.112537000	-2.586247000	-0.003386000

1	4.407222000	-1.805717000	-0.007792000
1	2.885579000	-3.767262000	0.005745000
6	-1.786912000	-2.179248000	-0.012654000
6	-3.687262000	-0.686175000	-0.005166000
6	-3.137414000	-1.985912000	-0.010118000
1	-3.812728000	-2.833744000	-0.012297000
6	5.093541000	0.590539000	-0.041504000
6	6.299841000	0.407557000	-0.046693000
14	8.101723000	0.041581000	-0.000247000
6	8.992255000	1.725667000	-0.339743000
6	8.421633000	-0.653024000	1.779880000
6	8.392739000	-1.275386000	-1.393326000
6	8.367820000	-0.620641000	-2.779419000
1	9.203998000	0.063432000	-2.939448000
1	8.437354000	-1.403395000	-3.546086000
1	7.436118000	-0.074083000	-2.953150000
6	7.258908000	-2.310024000	-1.389081000
1	7.430769000	-3.034708000	-2.195820000
1	7.190647000	-2.869944000	-0.456093000
1	6.291268000	-1.834327000	-1.570559000
6	9.734431000	-1.995418000	-1.221139000
1	9.882417000	-2.699252000	-2.050657000
1	10.581271000	-1.303318000	-1.226255000
1	9.780281000	-2.573459000	-0.295010000
6	7.872609000	-2.078122000	1.915841000
1	8.403421000	-2.799608000	1.291085000
1	7.984256000	-2.406287000	2.957397000
1	6.806800000	-2.125872000	1.671692000
6	9.915578000	-0.671740000	2.120892000
1	10.059774000	-1.110595000	3.116870000
1	10.498353000	-1.268305000	1.413606000
1	10.346118000	0.332502000	2.146620000
6	10.467969000	1.527634000	-0.703093000
1	10.942566000	2.505540000	-0.856995000
1	11.025833000	1.014735000	0.085411000
1	10.594056000	0.958099000	-1.627088000
6	7.679070000	0.193143000	2.823807000
1	7.836765000	-0.246583000	3.817468000
1	8.027162000	1.225190000	2.864509000
1	6.602965000	0.208643000	2.631486000
6	8.914797000	2.640833000	0.887986000
1	9.487082000	2.257601000	1.735790000
1	9.334363000	3.621890000	0.629721000
1	7.882074000	2.799929000	1.212568000
6	8.288364000	2.471872000	-1.481875000

1	8.773795000	3.446112000	-1.626152000
1	8.334080000	1.939971000	-2.432076000
1	7.235167000	2.653115000	-1.250072000
1	0.472633000	-3.457765000	0.003411000
1	-1.412369000	-3.193318000	-0.016995000
1	-0.461210000	3.541846000	-0.006059000
1	1.425441000	3.277013000	-0.038749000
1	-4.395894000	1.888470000	0.000500000
6	-5.081757000	-0.514397000	0.001125000
6	-6.290987000	-0.352530000	0.008035000
14	-8.107902000	-0.067731000	0.004435000
6	-8.340779000	1.686221000	0.794393000
6	-8.632758000	-0.145570000	-1.858692000
6	-8.857361000	-1.497323000	1.072569000
6	-8.602726000	-1.253368000	2.564701000
1	-9.139986000	-0.383277000	2.948304000
1	-8.950013000	-2.125952000	3.133291000
1	-7.537637000	-1.124202000	2.778763000
6	-8.172704000	-2.828830000	0.732452000
1	-8.583408000	-3.616695000	1.377565000
1	-8.325906000	-3.138643000	-0.301248000
1	-7.095391000	-2.777192000	0.912099000
6	-10.368341000	-1.640318000	0.855886000
1	-10.760997000	-2.423230000	1.517852000
1	-10.911999000	-0.718804000	1.082473000
1	-10.614879000	-1.931125000	-0.168369000
6	-8.633294000	-1.594673000	-2.358657000
1	-9.402042000	-2.207027000	-1.881949000
1	-8.836332000	-1.600702000	-3.437540000
1	-7.662692000	-2.075990000	-2.205158000
6	-10.027388000	0.448387000	-2.083204000
1	-10.304372000	0.346835000	-3.140621000
1	-10.796762000	-0.058257000	-1.494039000
1	-10.066771000	1.513187000	-1.840004000
6	-9.794224000	1.933692000	1.212605000
1	-9.891265000	2.947836000	1.621722000
1	-10.489200000	1.852373000	0.372352000
1	-10.124832000	1.239164000	1.988571000
6	-7.618749000	0.610627000	-2.728699000
1	-7.909933000	0.513954000	-3.782899000
1	-7.568816000	1.675399000	-2.500648000
1	-6.612891000	0.196305000	-2.619719000
6	-7.925817000	2.786428000	-0.189553000
1	-8.584725000	2.849350000	-1.058341000
1	-7.968535000	3.757371000	0.321172000

1	-6.900482000	2.648429000	-0.546842000
6	-7.431725000	1.840969000	2.021920000
1	-7.539620000	2.857880000	2.421863000
1	-7.675355000	1.146676000	2.825850000
1	-6.379746000	1.692805000	1.762962000

Table C7. PBE0-D3(BJ)/6-311G(d,p) optimized xyz Cartesian coordinates (Å) for the first singlet excited state (S_1) of **TIPS-PY**.

6	-3.690872000	-0.759782000	-0.049241000
6	-2.824685000	0.380284000	-0.028371000
6	-1.410389000	0.185059000	-0.030734000
6	-0.873529000	-1.130300000	-0.039174000
6	-1.770943000	-2.228809000	-0.060794000
6	-3.131886000	-2.047626000	-0.067334000
6	-0.549381000	1.321193000	-0.023309000
6	0.549446000	-1.320866000	-0.023739000
6	1.410453000	-0.184729000	-0.030903000
6	0.873594000	1.130632000	-0.038917000
6	2.824749000	-0.379956000	-0.028704000
6	3.343802000	-1.682580000	-0.006040000
6	2.495887000	-2.781772000	0.009071000
6	1.125956000	-2.611197000	-0.001576000
1	-3.797168000	-2.903099000	-0.086032000
1	2.913498000	-3.782599000	0.029049000
6	-3.343735000	1.682901000	-0.005285000
6	-2.495819000	2.782087000	0.010082000
6	-1.125888000	2.611516000	-0.000723000
1	-4.419604000	1.814829000	0.002604000
1	-2.913430000	3.782907000	0.030385000
6	1.771007000	2.229148000	-0.060298000
6	3.690935000	0.760115000	-0.049303000
6	3.131949000	2.047967000	-0.066994000
1	3.797231000	2.903445000	-0.085500000
6	-5.084519000	-0.592333000	-0.052992000
6	-6.292958000	-0.415904000	-0.054921000
14	-8.094321000	-0.059282000	0.001731000
6	-8.980508000	-1.739919000	-0.365610000
6	-8.412865000	0.601549000	1.796475000
6	-8.393895000	1.283077000	-1.366350000
6	-8.368324000	0.651430000	-2.763023000
1	-9.200232000	-0.035587000	-2.932336000
1	-8.445105000	1.446277000	-3.516469000
1	-7.433245000	0.114253000	-2.947282000
6	-7.265436000	2.323275000	-1.346628000

1	-7.441929000	3.059317000	-2.142073000
1	-7.199078000	2.869849000	-0.405492000
1	-6.295405000	1.855607000	-1.536439000
6	-9.738618000	1.993079000	-1.178626000
1	-9.891618000	2.711238000	-1.994902000
1	-10.582187000	1.297189000	-1.194651000
1	-9.784882000	2.553819000	-0.241997000
6	-7.863534000	2.023937000	1.956311000
1	-8.396848000	2.756914000	1.347204000
1	-7.971471000	2.332883000	3.004166000
1	-6.798542000	2.075816000	1.709329000
6	-9.906238000	0.613804000	2.139755000
1	-10.048495000	1.035227000	3.143623000
1	-10.490348000	1.222867000	1.444281000
1	-10.336841000	-0.390721000	2.149198000
6	-10.457493000	-1.540065000	-0.722765000
1	-10.929468000	-2.516666000	-0.892361000
1	-11.015492000	-1.042373000	0.075391000
1	-10.587137000	-0.955426000	-1.636819000
6	-7.668008000	-0.262898000	2.823505000
1	-7.822224000	0.160430000	3.824853000
1	-8.016995000	-1.295129000	2.848365000
1	-6.592544000	-0.276031000	2.627163000
6	-8.898292000	-2.674885000	0.846671000
1	-9.470310000	-2.307040000	1.701414000
1	-9.315467000	-3.652904000	0.573270000
1	-7.864550000	-2.836145000	1.166928000
6	-8.276672000	-2.465267000	-1.521228000
1	-8.758541000	-3.439189000	-1.679223000
1	-8.327150000	-1.919146000	-2.463151000
1	-7.222103000	-2.645722000	-1.294985000
1	-0.492401000	3.488154000	0.014921000
1	1.387455000	3.240155000	-0.077995000
1	0.492470000	-3.487839000	0.013863000
1	-1.387391000	-3.239810000	-0.078801000
1	4.419671000	-1.814510000	0.001733000
6	5.084585000	0.592680000	-0.053201000
6	6.293016000	0.416194000	-0.055210000
14	8.094298000	0.059171000	0.001544000
6	8.412580000	-0.601855000	1.796264000
6	8.393701000	-1.283159000	-1.366602000
6	8.980858000	1.739652000	-0.365605000
6	8.898823000	2.674508000	0.846774000
1	9.470745000	2.306449000	1.701490000
1	9.316216000	3.652465000	0.573483000

1	7.865109000	2.835957000	1.167030000
6	8.277200000	2.465276000	-1.521155000
1	8.759273000	3.439117000	-1.679023000
1	8.327585000	1.919256000	-2.463141000
1	7.222662000	2.645919000	-1.294915000
6	10.457808000	1.539515000	-0.722757000
1	10.929996000	2.516033000	-0.892241000
1	11.015683000	1.041620000	0.075361000
1	10.587346000	0.954942000	-1.636867000
6	8.368409000	-0.651427000	-2.763241000
1	9.200498000	0.035399000	-2.932438000
1	8.445066000	-1.446248000	-3.516725000
1	7.433477000	-0.114012000	-2.947553000
6	9.738246000	-1.993480000	-1.178802000
1	9.891157000	-2.711621000	-1.995110000
1	10.581969000	-1.297774000	-1.194712000
1	9.784311000	-2.554290000	-0.242205000
6	9.905932000	-0.614498000	2.139620000
1	10.048032000	-1.036015000	3.143471000
1	10.489928000	-1.223665000	1.444142000
1	10.336780000	0.389920000	2.149141000
6	7.264998000	-2.323095000	-1.347052000
1	7.441404000	-3.059139000	-2.142513000
1	7.198414000	-2.869693000	-0.405946000
1	6.295097000	-1.855188000	-1.536944000
6	7.862883000	-2.024110000	1.955997000
1	8.396024000	-2.757186000	1.346860000
1	7.970712000	-2.333146000	3.003837000
1	6.797885000	-2.075702000	1.708981000
6	7.667889000	0.262731000	2.823297000
1	7.821974000	-0.160659000	3.824640000
1	8.017110000	1.294882000	2.848196000
1	6.592433000	0.276119000	2.626924000

Table C8. PBE0-D3(BJ)/6-311G(d,p) optimized xyz Cartesian coordinates (Å) for the second triplet excited state (T_2) of **TIPS-PY**.

6	-3.715306000	0.784969000	0.055508000
6	-2.821630000	-0.362467000	0.011480000
6	-1.428295000	-0.162798000	0.018431000
6	-0.884655000	1.174495000	0.042896000
6	-1.795188000	2.261374000	0.098323000
6	-3.149996000	2.081015000	0.105664000
6	-0.557020000	-1.292036000	-0.000643000
6	0.543791000	1.377782000	0.004102000

6	1.415166000	0.248381000	0.012164000
6	0.871439000	-1.088951000	0.032624000
6	2.808576000	0.447736000	-0.000951000
6	3.335605000	1.752347000	-0.038222000
6	2.493206000	2.838228000	-0.062529000
6	1.110099000	2.653047000	-0.040819000
1	-3.815519000	2.935240000	0.150396000
1	2.899008000	3.843308000	-0.097539000
6	-3.348626000	-1.666911000	-0.034344000
6	-2.506072000	-2.752439000	-0.068995000
6	-1.123054000	-2.567187000	-0.050881000
1	-4.424929000	-1.794764000	-0.043547000
1	-2.911990000	-3.757280000	-0.109330000
6	1.781562000	-2.176356000	0.079714000
6	3.701695000	-0.700192000	0.026530000
6	3.136240000	-1.996371000	0.077501000
1	3.802163000	-2.850663000	0.114957000
6	-5.092265000	0.606666000	0.057350000
6	-6.305665000	0.418350000	0.059993000
14	-8.102914000	0.042907000	0.002430000
6	-9.004964000	1.719552000	0.348373000
6	-8.411462000	-0.642650000	-1.784742000
6	-8.392983000	-1.286334000	1.385117000
6	-8.376734000	-0.640583000	2.775462000
1	-9.215948000	0.039586000	2.936244000
1	-8.446983000	-1.428617000	3.536619000
1	-7.447707000	-0.091974000	2.956769000
6	-7.254116000	-2.315377000	1.377838000
1	-7.425350000	-3.046043000	2.179297000
1	-7.180262000	-2.869345000	0.441720000
1	-6.288841000	-1.837242000	1.565486000
6	-9.730394000	-2.011368000	1.202098000
1	-9.877907000	-2.722518000	2.025453000
1	-10.580733000	-1.323630000	1.209474000
1	-9.769652000	-2.582260000	0.271315000
6	-7.853967000	-2.063497000	-1.928383000
1	-8.383625000	-2.792987000	-1.311993000
1	-7.958638000	-2.384281000	-2.972967000
1	-6.789062000	-2.107046000	-1.679705000
6	-9.903829000	-0.666577000	-2.131715000
1	-10.041533000	-1.098457000	-3.131702000
1	-10.486129000	-1.271960000	-1.431569000
1	-10.339774000	0.335437000	-2.151502000
6	-10.480412000	1.509421000	0.705890000
1	-10.962069000	2.483152000	0.864111000

1	-11.032399000	0.997476000	-0.087327000
1	-10.605483000	0.933661000	1.626162000
6	-7.668642000	0.215372000	-2.818681000
1	-7.821612000	-0.216008000	-3.816740000
1	-8.019796000	1.246635000	-2.851409000
1	-6.593078000	0.232612000	-2.622952000
6	-8.929677000	2.642963000	-0.873229000
1	-9.495927000	2.260974000	-1.725628000
1	-9.357343000	3.619291000	-0.610394000
1	-7.896942000	2.811828000	-1.192815000
6	-8.309612000	2.462843000	1.497611000
1	-8.802782000	3.432312000	1.647685000
1	-8.353492000	1.923799000	2.443894000
1	-7.257311000	2.654064000	1.269692000
1	-0.487966000	-3.442712000	-0.083451000
1	1.403384000	-3.189108000	0.125061000
1	0.475175000	3.528975000	-0.065244000
1	-1.417111000	3.274180000	0.143559000
1	4.412176000	1.879441000	-0.047767000
6	5.079428000	-0.529448000	0.009215000
6	6.295901000	-0.363496000	-0.008874000
14	8.109115000	-0.069682000	-0.005344000
6	8.332365000	1.685669000	-0.795365000
6	8.635061000	-0.143631000	1.859261000
6	8.868459000	-1.496184000	-1.071168000
6	8.614267000	-1.254878000	-2.563799000
1	9.145743000	-0.381149000	-2.947166000
1	8.968731000	-2.125255000	-3.131325000
1	7.548601000	-1.133552000	-2.779506000
6	8.191100000	-2.831121000	-0.730351000
1	8.607254000	-3.617363000	-1.373928000
1	8.344668000	-3.138828000	0.303932000
1	7.113724000	-2.786155000	-0.911584000
6	10.380008000	-1.629529000	-0.852567000
1	10.778154000	-2.411018000	-1.512943000
1	10.918063000	-0.705005000	-1.080261000
1	10.627474000	-1.917155000	0.172345000
6	8.644589000	-1.592270000	2.359970000
1	9.416812000	-2.200411000	1.883508000
1	8.847972000	-1.596280000	3.438804000
1	7.676861000	-2.079563000	2.207195000
6	10.025798000	0.459566000	2.082230000
1	10.303790000	0.361043000	3.139694000
1	10.798184000	-0.042801000	1.493401000
1	10.057934000	1.524286000	1.837825000

6	9.784553000	1.940722000	-1.213575000
1	9.875866000	2.955091000	-1.623435000
1	10.479881000	1.864043000	-0.373192000
1	10.119308000	1.247569000	-1.989018000
6	7.616172000	0.607148000	2.728153000
1	7.909353000	0.516031000	3.782303000
1	7.557127000	1.670755000	2.496944000
1	6.613448000	0.184425000	2.621882000
6	7.911273000	2.784239000	0.187758000
1	8.569823000	2.851507000	1.056518000
1	7.948751000	3.755014000	-0.323696000
1	6.886658000	2.641182000	0.545115000
6	7.422847000	1.834221000	-2.023310000
1	7.526503000	2.850756000	-2.425308000
1	7.669398000	1.139271000	-2.825767000
1	6.371274000	1.682625000	-1.764532000

Table C9. PBE0-D3(BJ)/6-311G(d,p) optimized xyz Cartesian coordinates (Å) for the ground state (S_oS_o) of the **PY** dimer.

6	2.108516000	3.271017000	-0.858453000
6	3.714427000	-0.319134000	0.702663000
6	0.349861000	1.596545000	-1.202535000
6	1.445980000	0.100488000	1.558381000
6	-0.104342000	3.642478000	-0.001764000
6	3.230785000	1.717968000	1.898007000
6	1.954537000	-1.997531000	0.328416000
6	-1.954451000	1.998007000	-0.328889000
6	-0.349882000	-1.596663000	1.202123000
6	-1.446334000	-0.100289000	-1.558542000
6	-3.714497000	0.319555000	-0.702244000
6	-2.613415000	-1.190434000	2.054344000
6	-4.213805000	2.344468000	0.508731000
6	-0.886616000	0.445676000	2.373253000
1	5.157979000	1.231482000	1.103142000
1	1.534051000	5.020139000	0.221114000
1	-5.650097000	0.807494000	0.112358000
1	-2.917860000	0.675471000	3.041738000
6	2.613361000	1.189679000	-2.054551000
6	4.214093000	-2.343732000	-0.508705000
6	0.886086000	-0.445871000	-2.373742000
1	5.650274000	-0.806886000	-0.111449000
1	2.917275000	-0.676163000	-3.042223000
6	0.104801000	-3.642455000	0.001345000
6	-2.108018000	-3.271775000	0.858477000
1	-5.158069000	-1.231357000	-1.101485000
6	-3.231130000	-1.718032000	-1.896844000
1	-1.533031000	-5.020855000	-0.220922000

1	2.605355000	-3.724538000	-0.761378000
1	-1.224327000	-1.971570000	-2.576758000
1	-2.604962000	3.725265000	0.760714000
1	1.223778000	1.971256000	2.577486000
6	4.125589000	0.922640000	1.233738000
6	1.698978000	2.023956000	-1.376694000
6	2.362688000	-0.743079000	0.866533000
6	-0.563219000	2.435294000	-0.500661000
6	1.901813000	1.307584000	2.057394000
6	1.219930000	4.063058000	-0.181817000
6	-0.054511000	0.334538000	-1.724797000
6	0.054179000	-0.334556000	1.724388000
6	-2.362818000	0.743484000	-0.866666000
6	0.563383000	-2.435119000	0.500150000
6	-1.698843000	-2.024509000	1.376502000
6	-4.624775000	1.143546000	-0.005746000
6	-2.210784000	0.023718000	2.541432000
6	-2.888560000	2.767028000	0.343808000
1	3.139175000	3.580301000	-0.997529000
1	3.541774000	2.675771000	2.300996000
1	-3.639996000	-1.523077000	2.166401000
1	-4.912362000	2.980029000	1.042854000
6	4.624908000	-1.142951000	0.006229000
6	2.210376000	-0.024268000	-2.541852000
6	2.888829000	-2.766380000	-0.344209000
1	3.640058000	1.522004000	-2.166482000
1	4.912814000	-2.979146000	-1.042788000
6	-1.902262000	-1.307615000	-2.056905000
6	-4.125761000	-0.922469000	-1.232634000
1	-3.138512000	-3.581475000	0.997864000
6	-1.219269000	-4.063552000	0.181758000
1	-3.542135000	-2.676121000	-2.299136000
1	0.609824000	-1.418936000	-2.759082000
1	0.777564000	-4.297784000	-0.537849000
1	-0.610604000	1.418879000	2.758426000
1	-0.776944000	4.297955000	0.537447000

Table C10. PBE0-D3(BJ)/6-311G(d,p) optimized xyz Cartesian coordinates (Å) for the ground state (S_oT_1) of the **PY** dimer.

6	-2.192945000	-3.392094000	-0.607117000
6	-3.606261000	0.537020000	0.696433000
6	-0.471076000	-1.718355000	-1.105254000
6	-1.360719000	0.035785000	1.566530000
6	0.051325000	-3.734556000	0.185249000
6	-3.222987000	-1.477866000	1.963875000
6	-1.773946000	2.129557000	0.294820000
6	1.835385000	-2.074813000	-0.345735000
6	0.507518000	1.645860000	1.181611000
6	1.237047000	-0.022680000	-1.587353000

6	3.562078000	-0.390069000	-0.856840000
6	2.761283000	1.132261000	1.998512000
6	4.149978000	-2.410583000	0.366223000
6	0.970915000	-0.434736000	2.316406000
1	-5.122369000	-0.929836000	1.142107000
1	-1.554436000	-5.087371000	0.550764000
1	5.546254000	-0.851159000	-0.160316000
1	3.001021000	-0.764769000	2.944123000
6	-2.769017000	-1.388556000	-1.892189000
6	-4.001743000	2.535982000	-0.596409000
6	-1.111459000	0.283698000	-2.363524000
1	-5.508152000	1.077843000	-0.161897000
1	-3.146310000	0.429314000	-2.975692000
6	0.117956000	3.739116000	0.038431000
6	2.323588000	3.261315000	0.858690000
1	4.950134000	1.175352000	-1.357792000
6	2.958474000	1.648461000	-2.040979000
1	1.798841000	5.069342000	-0.147523000
1	-2.332963000	3.838570000	-0.872949000
1	0.928194000	1.883067000	-2.596696000
1	2.595356000	-3.797628000	0.754847000
1	-1.229038000	-1.807127000	2.650528000
6	-4.077325000	-0.664906000	1.268197000
6	-1.825581000	-2.173443000	-1.206476000
6	-2.238315000	0.905388000	0.856779000
6	0.486181000	-2.512255000	-0.417542000
6	-1.874610000	-1.129188000	2.107728000
6	-1.253576000	-4.153872000	0.086443000
6	-0.106215000	-0.475434000	-1.688632000
6	0.054578000	0.398466000	1.699399000
6	2.202125000	-0.834488000	-0.933852000
6	-0.375430000	2.527715000	0.494343000
6	1.873527000	2.016993000	1.349215000
6	4.518094000	-1.194538000	-0.208804000
6	2.315095000	-0.073377000	2.468270000
6	2.848505000	-2.846424000	0.306869000
1	-3.222927000	-3.723533000	-0.684413000
1	-3.580466000	-2.403748000	2.401318000
1	3.802072000	1.420268000	2.104089000
1	4.898625000	-3.020254000	0.861557000
6	-4.470587000	1.373400000	-0.042031000
6	-2.402501000	-0.171743000	-2.465165000
6	-2.664685000	2.911312000	-0.422823000
1	-3.793393000	-1.739679000	-1.956223000
1	-4.665123000	3.177045000	-1.167479000
6	1.652676000	1.233858000	-2.126121000
6	3.917693000	0.847363000	-1.422235000
1	3.366149000	3.529644000	0.995880000
6	1.455759000	4.109127000	0.222875000
1	3.243422000	2.611112000	-2.451259000
1	-0.858382000	1.236773000	-2.806136000

1	-0.537953000	4.435917000	-0.468544000
1	0.658907000	-1.402410000	2.687978000
1	0.757738000	-4.348933000	0.726824000

Table C11. PBE0-D3(BJ)/6-311G(d,p) optimized xyz Cartesian coordinates (Å) for the ground state (T_1T_1) of the **PY** dimer.

6	-2.106364000	-3.243996000	-0.900404000
6	-3.682578000	0.305715000	0.721884000
6	-0.346769000	-1.567801000	-1.230493000
6	-1.408488000	-0.112747000	1.575567000
6	0.097845000	-3.661854000	-0.036489000
6	-3.184421000	-1.755770000	1.914878000
6	-1.912349000	1.985298000	0.368518000
6	1.912985000	-1.984723000	-0.368690000
6	0.346913000	1.567127000	1.231022000
6	1.407731000	0.112813000	-1.576041000
6	3.682271000	-0.304340000	-0.722855000
6	2.610749000	1.152984000	2.075468000
6	4.171973000	-2.358802000	0.486493000
6	0.907175000	-0.506656000	2.410602000
1	-5.122061000	-1.244833000	1.115290000
1	-1.530790000	-5.020322000	0.166130000
1	5.612237000	-0.800708000	0.090743000
1	2.922048000	-0.731544000	3.061884000
6	-2.611049000	-1.154941000	-2.074396000
6	-4.170834000	2.360513000	-0.487481000
6	-0.908387000	0.505449000	-2.410363000
1	-5.612061000	0.803201000	-0.092186000
1	-2.923570000	0.729292000	-3.061014000
6	-0.096405000	3.661780000	0.037574000
6	2.107408000	3.242569000	0.901833000
1	5.120802000	1.246980000	-1.116706000
6	3.182734000	1.756684000	-1.916052000
1	1.532901000	5.019558000	-0.164187000
1	-2.583069000	3.741196000	-0.736775000
1	1.191372000	2.033583000	-2.582984000
1	2.585004000	-3.740315000	0.736287000
1	-1.193294000	-2.033853000	2.582101000
6	-4.090267000	-0.934910000	1.245901000
6	-1.702947000	-1.995071000	-1.406630000
6	-2.324829000	0.730511000	0.892407000
6	0.568953000	-2.407904000	-0.541006000
6	-1.877275000	-1.366892000	2.076985000
6	-1.202525000	-4.061548000	-0.221710000
6	0.061245000	-0.307283000	-1.743299000
6	-0.061839000	0.306655000	1.743336000
6	2.324697000	-0.729858000	-0.892972000
6	-0.568208000	2.407850000	0.541476000
6	1.703241000	1.993717000	1.407628000

6	4.586373000	-1.132297000	-0.032222000
6	2.203773000	-0.086470000	2.568701000
6	2.875234000	-2.782672000	0.326290000
1	-3.136514000	-3.555305000	-1.036335000
1	-3.510454000	-2.714947000	2.302728000
1	3.638927000	1.478774000	2.190320000
1	4.880928000	-2.986084000	1.016942000
6	-4.586047000	1.134213000	0.031077000
6	-2.204828000	0.084628000	-2.567978000
6	-2.873935000	2.783723000	-0.326835000
1	-3.639100000	-1.481264000	-2.188862000
1	-4.879287000	2.988157000	-1.018173000
6	1.875789000	1.367038000	-2.077913000
6	4.089167000	0.936445000	-1.247104000
1	3.137671000	3.553328000	1.038161000
6	1.204109000	4.060791000	0.223220000
1	3.508127000	2.716002000	-2.304090000
1	-0.625946000	1.478820000	-2.786598000
1	-0.774132000	4.318736000	-0.490273000
1	0.624143000	-1.479972000	2.786536000
1	0.775981000	-4.318277000	0.491497000

Table C12. PBE0-D3(BJ)/6-311G(d,p) optimized xyz Cartesian coordinates (Å) for the ground state (S_oS_o) of the **TIPS-PY** dimer.

6	-2.252819000	-3.013930000	-1.327112000
6	1.816936000	-2.198361000	1.747170000
6	-1.185994000	-0.803312000	-1.516731000
6	1.421863000	0.230693000	1.853002000
6	0.148609000	-2.817722000	-1.445356000
6	3.677438000	-0.644087000	1.655974000
6	-0.484973000	-1.355798000	1.896562000
6	1.302402000	-0.613609000	-1.561499000
6	-0.913208000	1.101782000	1.839199000
6	-0.094052000	1.437879000	-1.512935000
6	2.363707000	1.598791000	-1.466351000
6	-1.314777000	3.517010000	1.788297000
6	3.719554000	-0.388372000	-1.631114000
6	0.926483000	2.669046000	1.954934000
1	-0.913692000	-4.672675000	-1.237968000
1	0.414221000	4.755623000	1.967756000
6	-3.629263000	-0.972055000	-1.530188000
6	-0.028855000	-3.740815000	1.919456000
6	-2.568772000	1.180339000	-1.624730000
1	2.020476000	-4.339866000	1.735407000
1	-4.694384000	0.870591000	-1.684548000
6	-2.790097000	-0.420942000	1.792733000
6	-3.211853000	1.945256000	1.599160000
6	0.997378000	3.600739000	-1.344871000

1	-4.735646000	0.452524000	1.548075000
6	4.090273000	-3.003431000	1.261991000
6	-4.452394000	-4.362155000	-0.818512000
14	5.730779000	-5.247055000	0.017955000
6	-5.837720000	-6.903181000	0.050806000
6	-6.236047000	-4.049947000	1.533721000
6	-7.391604000	-4.498241000	-1.455814000
6	4.925640000	-7.362714000	-1.769445000
1	-7.633723000	-6.460414000	-2.421901000
1	-8.049809000	-5.074124000	-3.427555000
1	-6.351734000	-5.457919000	-3.126386000
6	3.216623000	-5.596711000	-1.320574000
1	-7.881577000	-2.840005000	-2.749248000
1	-7.157073000	-2.315204000	-1.229510000
1	-6.143214000	-3.034944000	-2.486676000
6	3.731576000	-7.259959000	0.409708000
1	-9.538762000	-4.314560000	-1.643303000
1	-9.054234000	-5.529010000	-0.456816000
1	-8.941437000	-3.809635000	-0.063593000
6	6.013749000	-6.273445000	2.662962000
1	-7.559052000	-2.437355000	0.832013000
1	-6.583378000	-2.042169000	2.245802000
1	-5.829352000	-2.090509000	0.647264000
6	7.366089000	-7.448014000	0.918655000
1	-7.427449000	-4.079670000	3.340013000
1	-8.306628000	-4.669734000	1.927356000
1	-7.093567000	-5.690959000	2.707154000
6	7.865786000	-5.233208000	-1.924076000
1	-7.010703000	-8.686374000	0.404413000
1	-7.715881000	-7.242736000	1.135957000
1	-7.843335000	-7.506363000	-0.606421000
6	8.011368000	-5.187259000	1.733990000
1	-5.009727000	-3.427611000	3.200715000
1	-4.615065000	-5.053074000	2.641925000
1	-4.097443000	-3.657530000	1.696637000
6	7.385933000	-3.058427000	-0.788065000
1	-5.394541000	-6.951751000	2.202992000
1	-4.742944000	-8.301744000	1.275672000
1	-3.967890000	-6.716181000	1.175444000
6	5.847568000	-3.901318000	-2.505046000
1	-4.964759000	-8.560102000	-1.025152000
1	-5.729167000	-7.386518000	-2.097064000
1	-4.170751000	-7.012050000	-1.358329000
1	-1.980580000	-2.886734000	2.027116000
1	-1.107119000	3.327867000	-1.433901000
1	2.683408000	-2.252632000	-1.662886000
1	3.207045000	1.421239000	1.795053000
1	4.514641000	1.595383000	-1.511780000
6	3.417093000	3.784204000	-1.100953000
6	-4.792078000	3.911824000	0.862578000
14	6.043298000	4.991033000	-0.154308000

6	-6.822023000	6.088624000	1.372528000
6	-4.358389000	6.415815000	-0.704306000
6	-6.755876000	4.341320000	-1.352475000
6	4.931183000	7.210148000	1.261502000
1	-8.110127000	3.248462000	0.002424000
1	-7.906413000	2.530206000	-1.595618000
1	-6.619795000	2.387239000	-0.385079000
6	5.137252000	7.492054000	-1.171166000
1	-6.425933000	3.295499000	-3.212002000
1	-5.433696000	4.744285000	-3.064518000
1	-5.013319000	3.285611000	-2.150474000
6	7.176932000	7.608327000	0.272716000
1	-8.390221000	4.698996000	-2.724871000
1	-8.608469000	5.502437000	-1.170875000
1	-7.471697000	6.160094000	-2.352933000
6	7.342406000	5.449027000	-2.665282000
1	-5.737429000	7.983588000	-1.384848000
1	-4.131649000	8.040118000	-2.116380000
1	-5.295697000	6.826432000	-2.644970000
6	8.801490000	4.547786000	-0.863355000
1	-2.890151000	7.834474000	-0.004460000
1	-4.429349000	7.978112000	0.842198000
1	-3.333589000	6.637986000	1.219148000
6	7.323536000	4.650172000	2.414967000
1	-8.011362000	7.865400000	1.701035000
1	-6.565561000	8.173524000	0.740972000
1	-7.960779000	7.376273000	0.005431000
6	7.118693000	3.096626000	-2.008289000
1	-2.431013000	6.283011000	-1.670000000
1	-2.766282000	4.925836000	-0.582082000
1	-3.527219000	4.997684000	-2.175300000
6	6.578177000	2.565595000	1.272217000
1	-5.174760000	6.963546000	2.547679000
1	-6.667380000	6.682059000	3.443023000
1	-5.613588000	5.321354000	3.024547000
6	4.906977000	4.037715000	2.299961000
1	-8.554625000	5.635251000	2.575309000
1	-8.718806000	5.073870000	0.912448000
1	-7.681322000	4.192149000	2.049271000
6	3.214505000	-1.944512000	1.597971000
6	-2.366673000	-1.595417000	-1.462755000
6	0.916105000	-1.100762000	1.838924000
6	0.091094000	-1.434588000	-1.511833000
6	2.792938000	0.421811000	1.790953000
6	-1.000267000	-3.597279000	-1.341100000
6	-1.305427000	0.616880000	-1.560746000
6	0.487978000	1.356895000	1.895182000
6	1.182978000	0.806611000	-1.518549000
6	-1.418969000	-0.229654000	1.854271000
6	-1.814196000	2.199277000	1.747447000
6	3.626245000	0.975404000	-1.534674000

6	0.031735000	3.741862000	1.916661000
6	2.565696000	-1.177071000	-1.626200000
1	4.738380000	-0.451822000	1.545937000
1	4.691228000	-0.867391000	-1.688304000
6	1.317515000	-3.516066000	1.789258000
6	-3.722635000	0.391645000	-1.627791000
6	-0.923405000	-2.667856000	1.958074000
1	-4.517692000	-1.591914000	-1.505551000
1	-0.411362000	-4.754501000	1.971841000
6	-0.151549000	2.821097000	-1.447538000
6	2.249933000	3.017343000	-1.331466000
6	-3.674692000	0.644837000	1.657835000
1	0.910927000	4.676185000	-1.242175000
6	-3.419784000	-3.781015000	-1.096228000
6	4.794714000	-3.911509000	0.862363000
14	-6.045663000	-4.992908000	-0.154143000
6	6.758358000	-4.341006000	-1.352842000
6	6.824548000	-6.088278000	1.372176000
6	4.361006000	-6.415571000	-0.704757000
6	-7.344852000	-5.436390000	-2.667912000
1	5.739934000	-7.983416000	-1.385263000
1	4.134280000	-8.039600000	-2.117109000
1	5.298603000	-6.826003000	-2.645305000
6	-7.120022000	-3.087808000	-1.998311000
1	2.433464000	-6.282574000	-1.670123000
1	2.769253000	-4.925059000	-0.582812000
1	3.530014000	-4.997771000	-2.176044000
6	-8.803462000	-4.544090000	-0.861094000
1	2.893029000	-7.834565000	-0.005082000
1	4.432143000	-7.977695000	0.841840000
1	3.335906000	-6.637862000	1.218517000
6	-6.575380000	-2.575028000	1.285660000
1	5.177346000	-6.963038000	2.547516000
1	6.670100000	-6.681696000	3.442676000
1	5.616370000	-5.320891000	3.024344000
6	-7.328566000	-4.663902000	2.415775000
1	8.013851000	-7.865125000	1.700727000
1	6.567904000	-8.173225000	0.740865000
1	7.963092000	-7.376142000	0.005076000
6	-7.180908000	-7.611993000	0.256306000
1	8.392792000	-4.698549000	-2.725139000
1	8.610854000	-5.502283000	-1.171258000
1	7.474159000	-6.159666000	-2.353524000
6	-4.910218000	-4.058611000	2.307729000
1	8.557162000	-5.634721000	2.574835000
1	8.721345000	-5.073743000	0.911824000
1	7.683909000	-4.191694000	2.048458000
6	-4.935803000	-7.221351000	1.249045000
1	8.112745000	-3.248173000	0.001969000
1	7.908974000	-2.529986000	-1.596085000
1	6.622458000	-2.386833000	-0.385467000

6	-5.140084000	-7.488134000	-1.185532000
1	6.428397000	-3.294969000	-3.212254000
1	5.436285000	-4.743859000	-3.065024000
1	5.015700000	-3.285352000	-2.150824000
1	-2.686514000	2.255880000	-1.662088000
1	-3.204035000	-1.420489000	1.796503000
1	1.983708000	2.888642000	2.022350000
1	1.104157000	-3.324538000	-1.432011000
1	-2.017815000	4.340749000	1.734695000
6	-4.087665000	3.003996000	1.262813000
6	4.450154000	4.364133000	-0.822425000
14	-5.728347000	5.247304000	0.018338000
6	6.233964000	4.038078000	1.527949000
6	7.389680000	4.504295000	-1.458316000
6	5.834348000	6.899936000	0.062087000
6	-7.383366000	3.058743000	-0.787660000
1	5.388696000	6.934242000	2.214230000
1	4.738174000	8.290325000	1.295076000
1	3.963376000	6.705438000	1.183310000
6	-5.845088000	3.901703000	-2.504677000
1	4.960427000	8.562730000	-1.003963000
1	5.727460000	7.397169000	-2.082704000
1	4.168683000	7.015931000	-1.348103000
6	-7.863308000	5.233545000	-1.923671000
1	7.005996000	8.681749000	0.426816000
1	7.711496000	7.234364000	1.150609000
1	7.839988000	7.507986000	-0.590170000
6	-4.922970000	7.363064000	-1.768958000
1	7.631224000	6.471730000	-2.413698000
1	8.047211000	5.091049000	-3.427098000
1	6.349149000	5.472931000	-3.123337000
6	-3.728937000	7.260141000	0.410223000
1	9.536888000	4.322213000	-1.646621000
1	9.051887000	5.530891000	-0.454461000
1	8.939760000	3.809696000	-0.069268000
6	-7.363661000	7.448277000	0.918918000
1	7.424135000	4.059971000	3.335215000
1	8.301864000	4.663088000	1.927176000
1	7.084498000	5.674290000	2.713273000
6	-3.213913000	5.597035000	-1.320090000
1	7.879364000	2.853768000	-2.761737000
1	7.157787000	2.319837000	-1.243834000
1	6.141148000	3.045360000	-2.495375000
6	-6.011085000	6.273880000	2.663223000
1	7.562839000	2.433313000	0.819163000
1	6.586561000	2.027618000	2.229514000
1	5.834443000	2.082237000	0.630327000
6	-8.008803000	5.187635000	1.734561000
1	5.008941000	3.405957000	3.192124000
1	4.604645000	5.029759000	2.634951000
1	4.098058000	3.632291000	1.686538000
