# Supplementary material for: Enhancing the statistical probability factor in triplet–triplet annihilation photon upconversion via TIPS functionalization
Source: Chem Sci. 2025 Oct 7;16(43):20255–64. doi: 10.1039/d5sc05248c (PMC12519238; doi:10.1039/d5sc05248c)
Supplement: SC-016-D5SC05248C-s002 [file SC-016-D5SC05248C-s002.pdf]

Supplementary Information (SI) for Chemical Science.
This journal is © The Royal Society of Chemistry 2025

Supporting information-I for

Enhancing the Statistical Probability Factor in Triplet-Triplet Annihilation Photon Upconversion via TIPS Functionalization

Lukas Naimovičius,[a,b,c] Manvydas Dapkevičius,[b] Edvinas Radiunas [b] Mila Miroshnichenko,[a] Gediminas Kreiza [b] Carles Alcaide,[d] Paulius Baronas,[e] Yoichi Sasaki,[f] Nobuhiro Yanai,[f] Nobuo Kimizuka,[f] Andrew B. Pun,[c] Miquel Solà,[d] Pankaj Bharmoria,*[a] Karolis Kazlauskas,[b] and Kasper Moth-Poulsen*[a,e,g,h]

[a] Institute of Materials Science of Barcelona, ICMAB-CSIC, Bellaterra, Barcelona, 08193, Spain.
[b] Institute of Photonics and Nanotechnology, Vilnius University, Saulėtekio av. 3, LT-10257 Vilnius, Lithuania.
[c] Department of Chemistry and Biochemistry, University of California San Diego, 92093 La Jolla, CA, USA.
[d] Institute of Computational Chemistry and Catalysis (IQCC), Universitat de Girona, M. Aurèlia Capmany 69, 17003 Girona, Spain.
[e] Department of Chemical Engineering, Universitat Politècnica de Catalunya, EEBE, Eduard Maristany 10–14, 08019 Barcelona, Spain.
[f] Department of Applied Chemistry, Graduate School of Engineering, Kyushu University, 744 Moto-oka, Nishi-ku, Fukuoka 819-0395, Japan.
[g] Catalan Institution for Research & Advanced Studies, ICREA, Pg. Lluís Companys 23, Barcelona, Spain.
[h] Department of Chemistry and Chemical Engineering, Chalmers University of Technology, Kemivagen 4, Gothenburg 412 96, Sweden.

*E-mails – kasper.moth-poulsen@upc.edu; pbharmoria@icmab.es

Contents

Annexure 1……………………………………………………………………………………………2

Synthesis procedure of 3,9-bis((triisopropylsilyl)ethynyl)perylene (TIPS-PY)………………………… 2-3

NMR Characterization of TIPS-PY……………………………………………………………3-4

MALDI-TOF analysis of TIPS-PY……………………………………………………………5

X-Ray Diffraction Analysis of TIPS-PY………………………………………………………5-6

Photophysical Measurements…………………………………………………………………6

Absorption spectra and extinction coefficient of TIPS-PY ……………………………………6

Fluorescence transients, digital images and spectra of TIPS-PY…………………………………7-8

DFT calculations………………………………………………………………………………8-14

Upconversion emission images of PdTPBP-TIPS-PY system ……………………………………14

Upconversion threshold of PdTPBP-TIPS-PY system……………………………………………15

Fluorescence spectra of TIPS-PY………………………………………………………………16

Absorption Spectra of TIPS-PY 16
TTA-UC Transients of PdTPBP-TIPS-PY system 17
Phosphorescence spectra of PdTPBP........18
UC quantum yield and UC transients of PdDPBP-BPEA system........19
Phosphorescence transients of Os(m-peptpy)2(TFSI)$_2$........20
Upconversion transients and spectra of TIPS-PY- Os(m-peptpy)2(TFSI)$_2$........ 21
Experimental........22
Materials 22
Optical Techniques........22
References........23

Annexure 1.

Synthesis procedure of 3,9-bis((triisopropylsilyl)ethynyl)perylene (TIPS-PY)

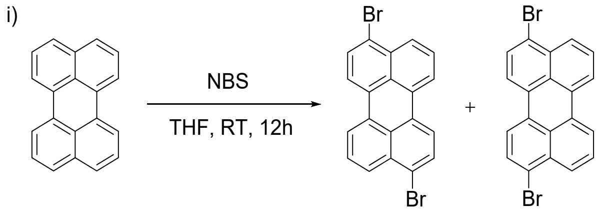

Perylene (**1**) was brominated using well known procedure using N-bromosuccinimide as a brominating agent in THF solution at room temperature.[1] Perylene (2 g, 7.94 mmol) was dissolved in THF (320 ml) in a 500 ml round bottom flask. N-bromosuccinimide (4.24 g, 23.8 mmol) was added in one portion after 10 min. Mixture allowed to stir overnight. The reaction was quenched by slowly pouring organics onto ice (1.5 l) while stirring. The dark green residue comprising a mixture of 3,9 and 3,10 dibromoperylene was washed with water multiple times, collected, and dried under vacuum for further synthesis (2.4 g, 5.85 mmol). The percent yield was 74%. The ^{1}H NMR spectra of the mixture recorded in $CDCl_3$ showed a similarity in chemical shifts corresponding to the one reported in literature for 3,9-dibromoperylene (**2**).[2]

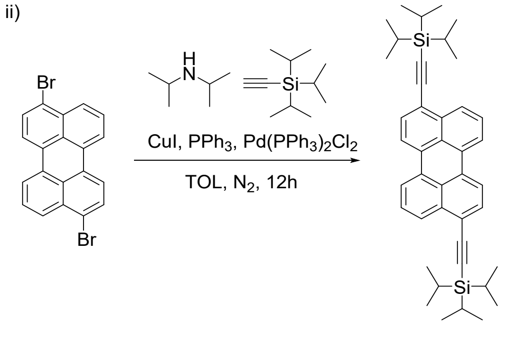

The final compound, 3,9-bis((triisopropylsilyl)ethynyl)perylene (**TIPS-PY**) was synthesized via Sonogashira coupling. Compound **2** (500 mg, 1 eq, 1.22 mmol), $Pd(PPh_3)_2Cl_2$ (18.83mg, 0.02 eq), CuI

(14.63mg, 0.06 eq) and PPh_3 (19.83 mg, 0.06eq) were dissolved in toluene (30 ml) under N_2 atmosphere. The mixture was heated to 100°C and diisopropylamine (5 ml) was added. Triisopropylsilylacetylene (1.2 ml) was then added dropwise. The mixture was left to stir overnight at 100°C under N_2. The reaction was monitored by TLC (30% EtOac in Hexane). The mixture was cooled to RT, and toluene and diisopropylamine were then removed *in vacuo*. The residue was purified by column chromatography with hexane as the eluent to yield **TIPS-PY** as an orange solid (160 mg, 0.26 mmol) with 21% yield. An image of the synthesized **TIPS-PY** is shown as Fig. S1.

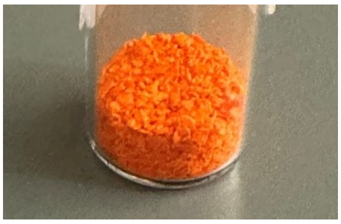

Fig S1. Synthesized and purified **TIPS-PY.**

NMR Characterization

^{1}H NMR and ^{13}C {^{1}H} NMR Spectroscopy: The ^{1}H NMR and ^{13}C {^{1}H} NMR spectra were acquired using a Bruker, 300 MHz NMR spectrometer including a Nanobay AVANCE nanoNEO console, ultra-shielded ASCEND magnet and a BBFO probe head. The chemical shifts and ^{1}H NMR and ^{13}C {^{1}H} NMR spectra are provided below.

^{1}H NMR of **TIPS-PY** (300 MHz,CDCl3): δ 8.21-8.30 (m, J = 8.26 Hz, 4H), 8.09-8.14 (dd, J = 8.12 Hz, 2H), 7.73-7.70 (dd, J = 7.72 Hz, 2H), 7.63-7.57 (ddd, J = 7.60Hz, 2H), 1.21-1.25(d, J= 1.24 Hz 42H).

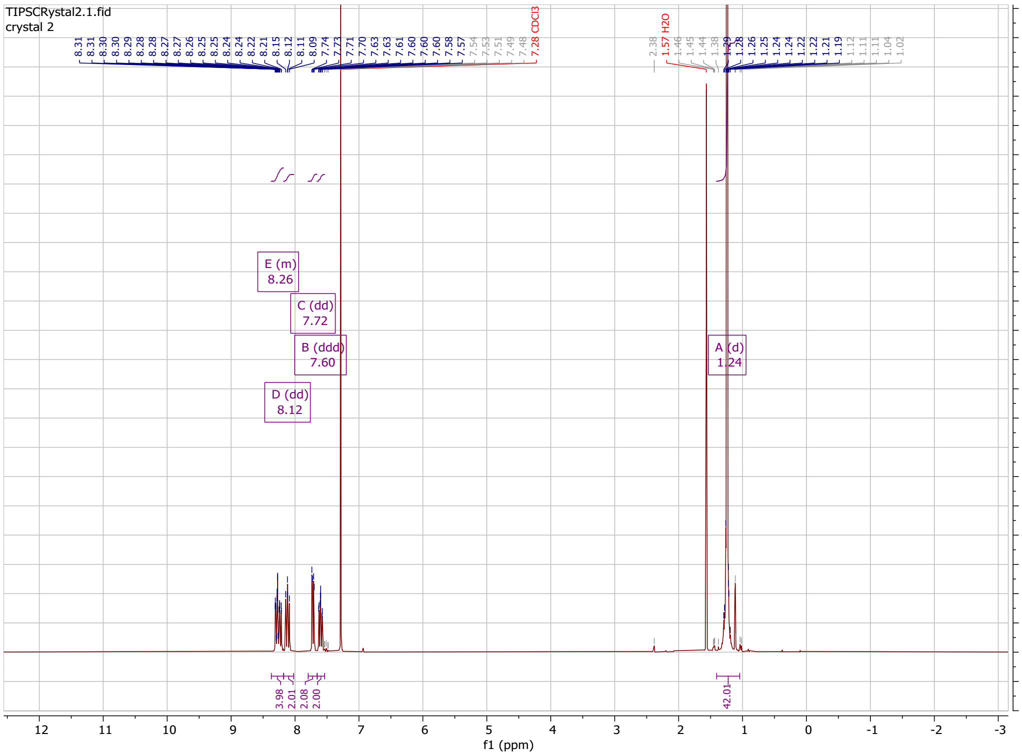

Fig. S2. 1H NMR spectrum of **TIPS-PY** in $CDCl_3$.

^{13}C {^{1}H} NMR of TIPS-PY (300MHz, $CDCl_3$) δ 134.61, 131.59, 131.50, 131.41, 131.07, 130.73, 127.99, 127.22, 127.14, 126.61, 126.30, 121.20, 120.95, 120.87, 120.65, 120.03, 119.71, 105.42, 105.39, 97.37, 97.12,18.85, 11.50.

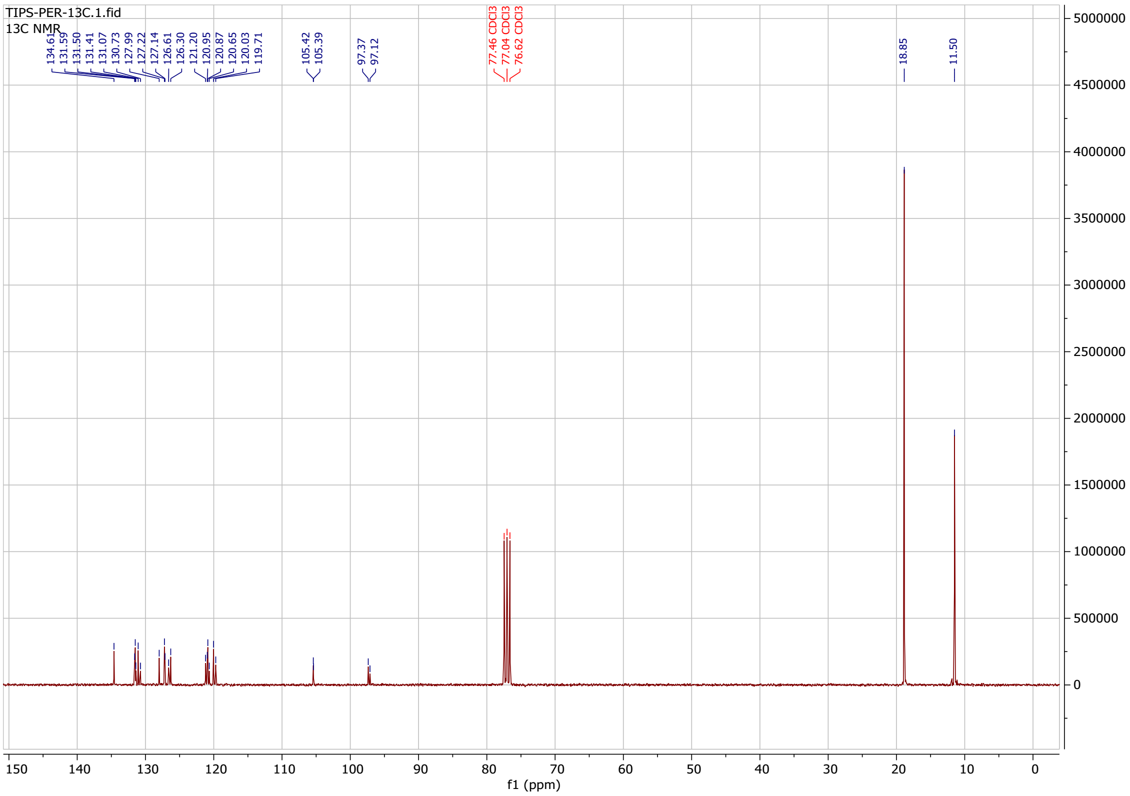

Fig. S3. ^{13}C {^{1}H} NMR) spectrum of **TIPS-PY** in $CDCl_3$.

MALDI-TOF analysis

Matrix-assisted laser desorption/ionization time of flight mass spectrometry (MALDI-TOF) analysis of the **TIPS-PY** was carried out using a Bruker Daltonics, Autoflex maX spectrophotometer equipped with Flex Control 3.4 and Flex Analysis 3.4 softwares.

The MALDI spectra of **TIPS-PY** showed a single peak corresponding to the exact mass 612.3 corresponding to 3,9-**TIPS-PY** Fig. S4).

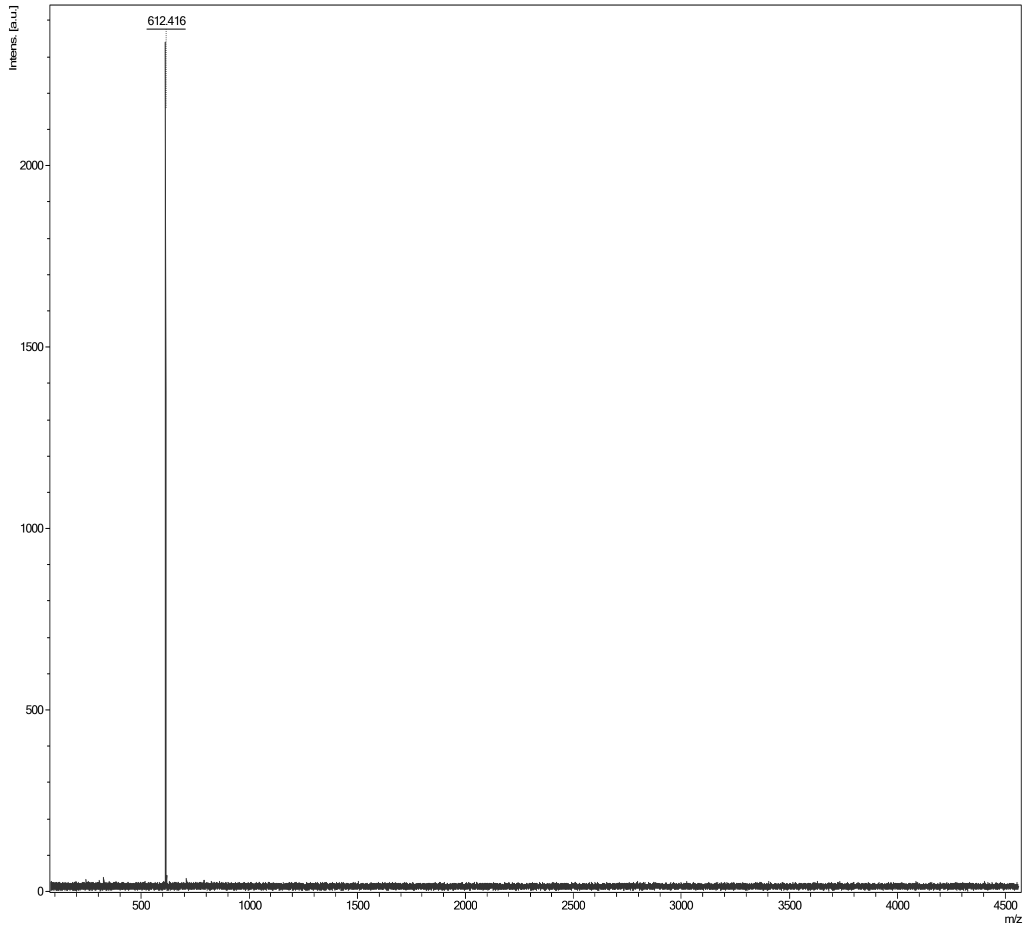

Fig. S4. MALDI-TOF spectra of **TIPS-PY**.

X-Ray Diffraction Analysis

Single crystals of TIPS-PY were grown by slow evaporation of THF solution. A suitable crystal was selected under a microscope and mounted on a micromount (MiTeGen) with inert oil used as adhesive and analyzed on a Rigaku XtaLAB Synergy-S diffractometer with HyPix-6000HE hybrid photon counting detector and PhotonJet microfocus X-ray source providing CuK_{α} (λ = 1.54184 Å) radiation. XRD measurements were performed at room temperature. Data were collected and integrated by CrysAlisPro software. The structure was solved using Intrinsic Phasing by the ShelXT program[3] and refined with the ShelXL package[4] using Least Squares minimization through Olex2 graphical interface.[5]

CCDC 2296125 contains the crystallographic data for TIPS-PY. XRD data is available via www.ccdc.cam.ac.uk/data_request/cif, or by emailing data_request@ccdc.cam.ac.uk, or by contacting The Cambridge Crystallographic Data Centre, 12 Union Road, Cambridge CB2 1EZ, UK; fax: +44 1223 336033.

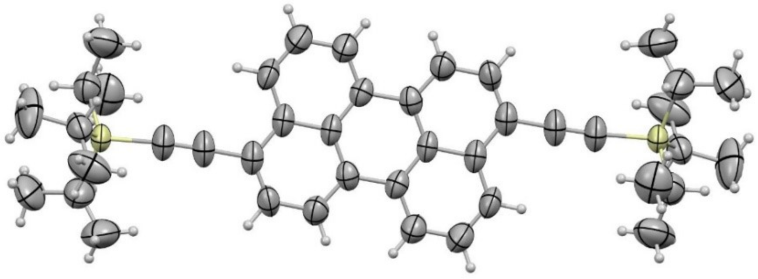

Fig. S5. Molecular structure of **TIPS-PY** obtained by single crystal X-Ray diffraction analysis.

Photophysical Measurements

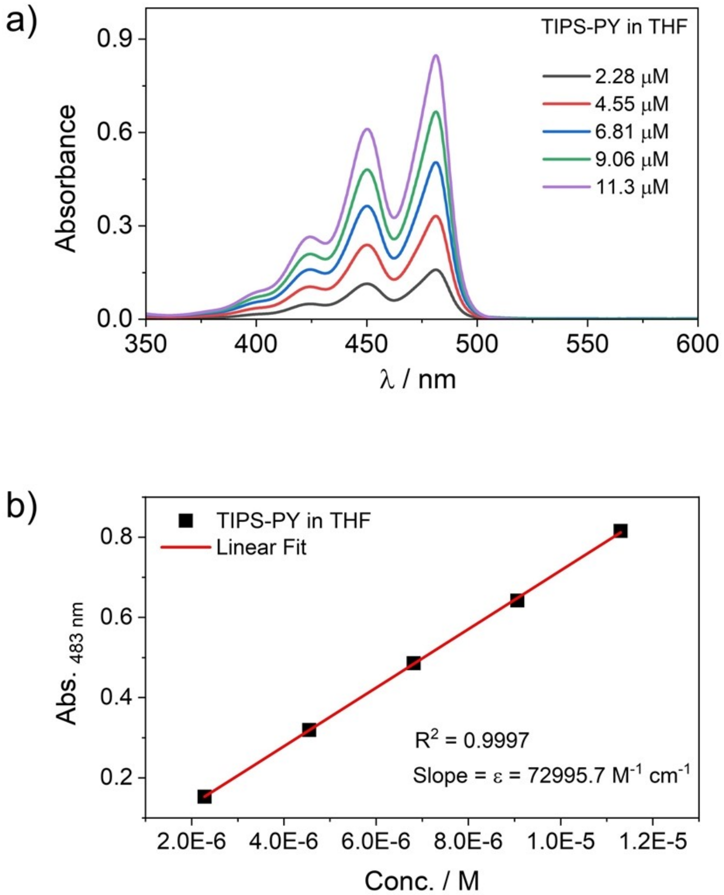

Fig. S6. a) Concentration dependent absorption spectra of **TIPS-PY** in THF. b) Beer-Lambert plot of **TIPS-PY** in THF. Extinction coefficient indicated.

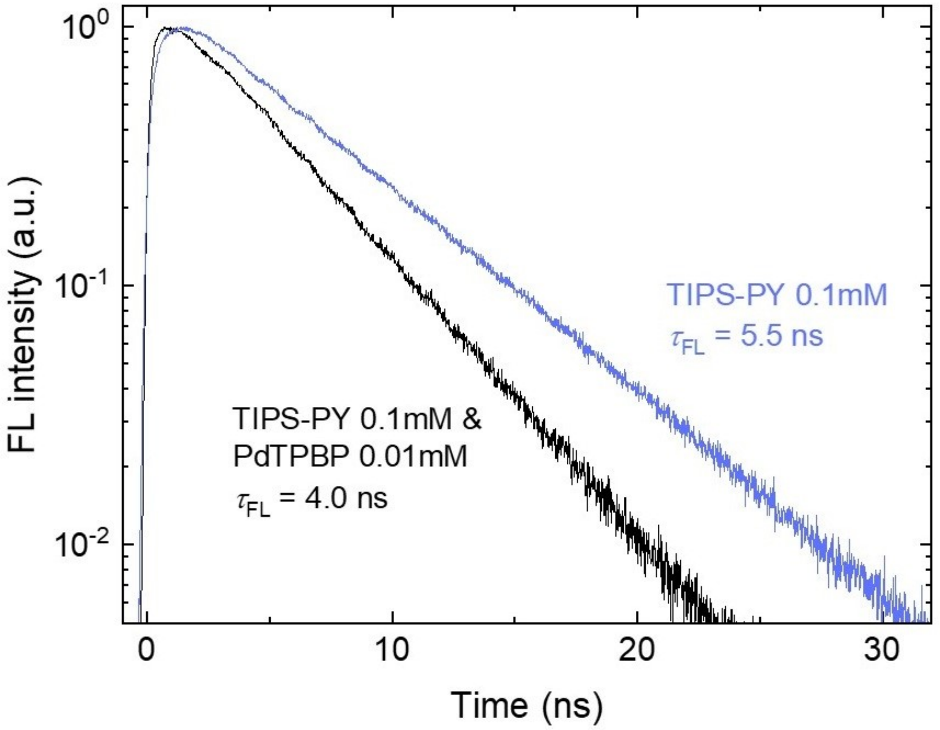

Fig. S7. Fluorescence transients (λ_{FL} = 489 nm) of **TIPS-PY** and **TIPS-PY:PdTPBP** UC solution at **TIPS-PY** concentration of 10^{-4} M and 10^{-5} M, respectively, in THF. Fluorescence lifetimes indicated.

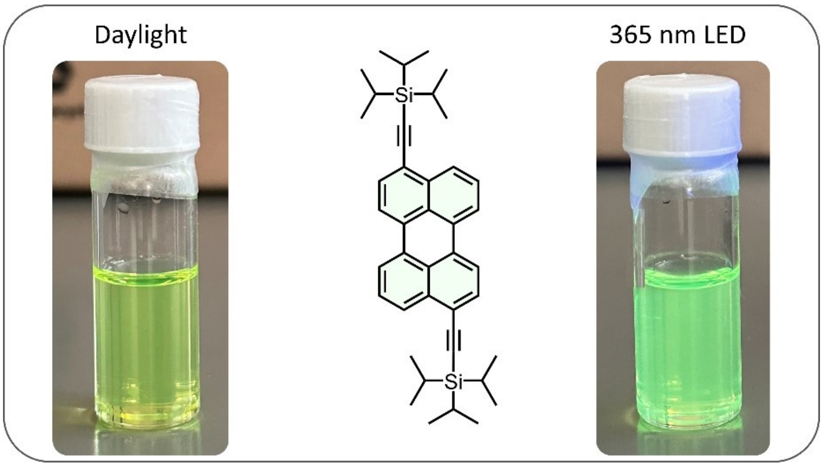

Fig. S8. Digital images of TIPS-PY solution at 10^{-5} M in THF under day light and 365 nm LED showing green emission.

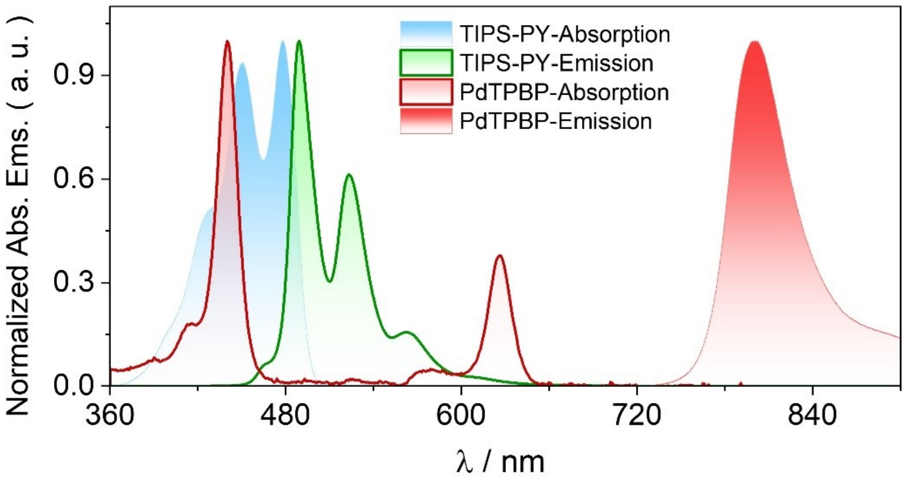

Fig. S9. Normalized absorption and emission spectra of **TIPS-PY** (annihilator, λ_{ex} = 420 nm CW laser) and **PdTPBP** (sensitizer, λ_{ex} = 640 nm CW laser) in THF.

DFT calculations

Full geometry optimizations have been carried out with the PBE0[6] density functional including dispersion corrections[7–9] with the 6-311G(d,p) basis set[10] and the Gaussian 16 program.[11] For all species, we have analyzed the lowest-lying closed-shell singlet ground state (S_0), the lowest-lying singlet excited state (S_1), and the lowest-lying triplet excited states (T_1 and T_2). For the latter, the geometry optimizations were performed within the unrestricted methodology, while for the former the restricted formalism was used. S_0 and T_1 states were optimized with the density functional theory (DFT) method, whereas S_1 and T_2 were optimized with the time-dependent DFT. All optimized stationary points were verified by performing a vibrational analysis calculation, to be energy minima (no imaginary frequencies).

For the calculation of the adiabatic singlet-triplet energy gap, ΔE_{ST}, we used several functionals with different percentages of Hartree-Fock exchange and the best results as compared to experimental values for anthracene, 9,10-diphenylanthracene, and 9,10-bis[tri-tert-butylsilyl)ethynil]anthracene were obtained with the PBE0[6] and B3LYP[12,13] functionals (Table S1). Finally, we chose the (U)PBE0-D3(BJ)/6-311G(d,p) method for the calculation of the reported ΔE_{ST}. Figure 2 (main manuscript) provides the triplet state spin density distribution in anthracene (**An**), **TIPS**-anthracene (**TIPS-An**), naphthalene (**Naph**), and **TIPS**-naphthalene (**TIPS-Naph**) chromophores.

The character of the S_0S_1 excited state was analysed with TheoDORE,[14] a fragment-based analysis for assigning state character.

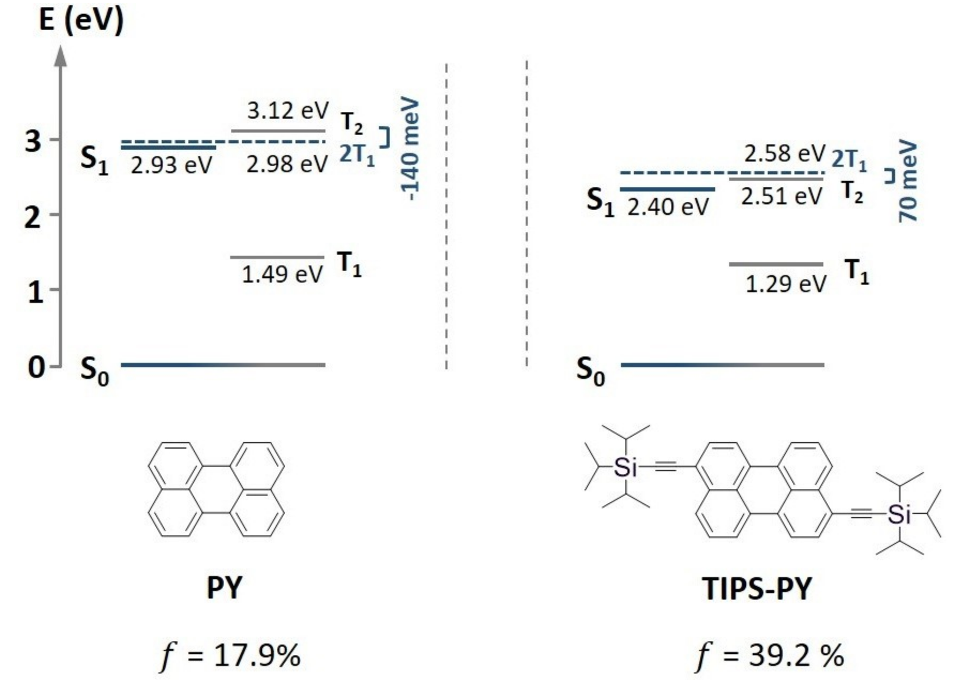

Fig. S10. Calculated excited energy states of **PY** and **TIPS-PY** chromophores

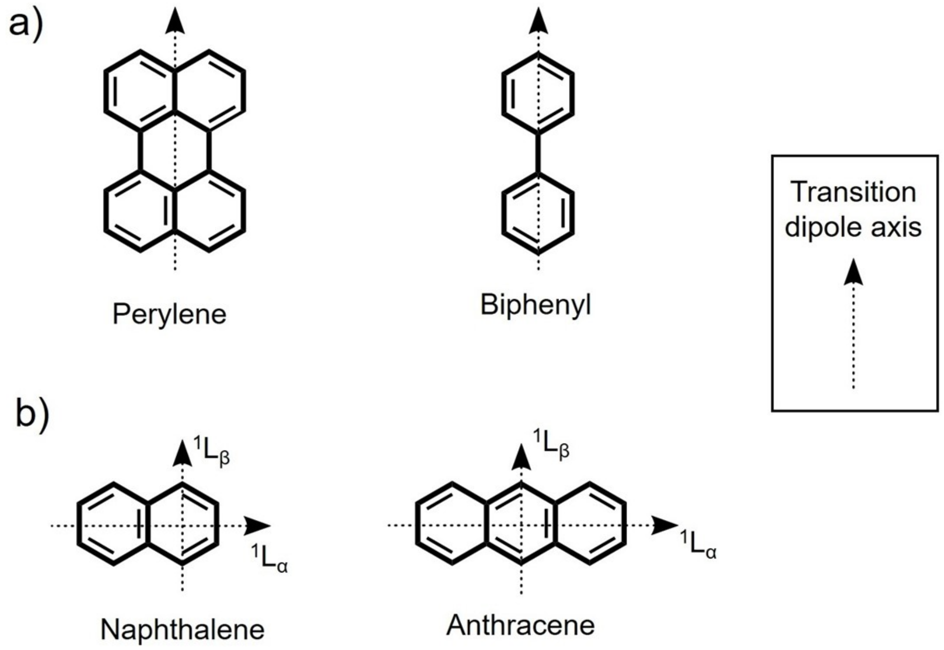

Fig. S11. Molecular structures of chromophores along with their transition dipole axis. a, b) perylene and biphenyl and c, d) naphthalene and anthracene.

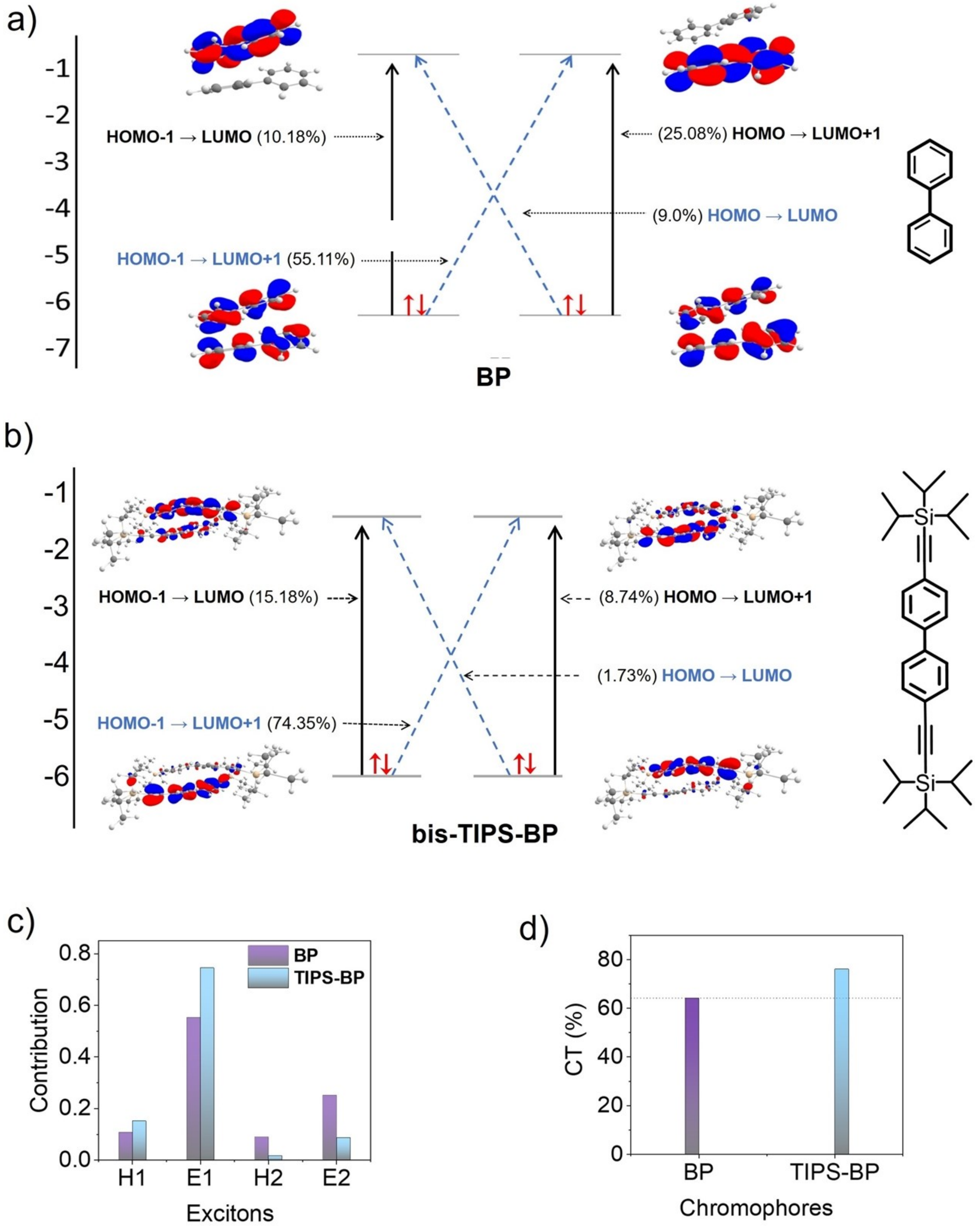

Fig. S12. a,b) Illustration of the contribution of a linear combination of orbital replacement in **BP** (a) and **TIPS-BP** (b) c) Plot showing comparative electron (E), and hole (H) contribution during orbital replacement in **BP** and **TIPS-BP** (energies in eV). d) Percentage of charge transfer (CT) calculated from E and H contribution during orbital replacement in **BP** and **TIPS-BP**.

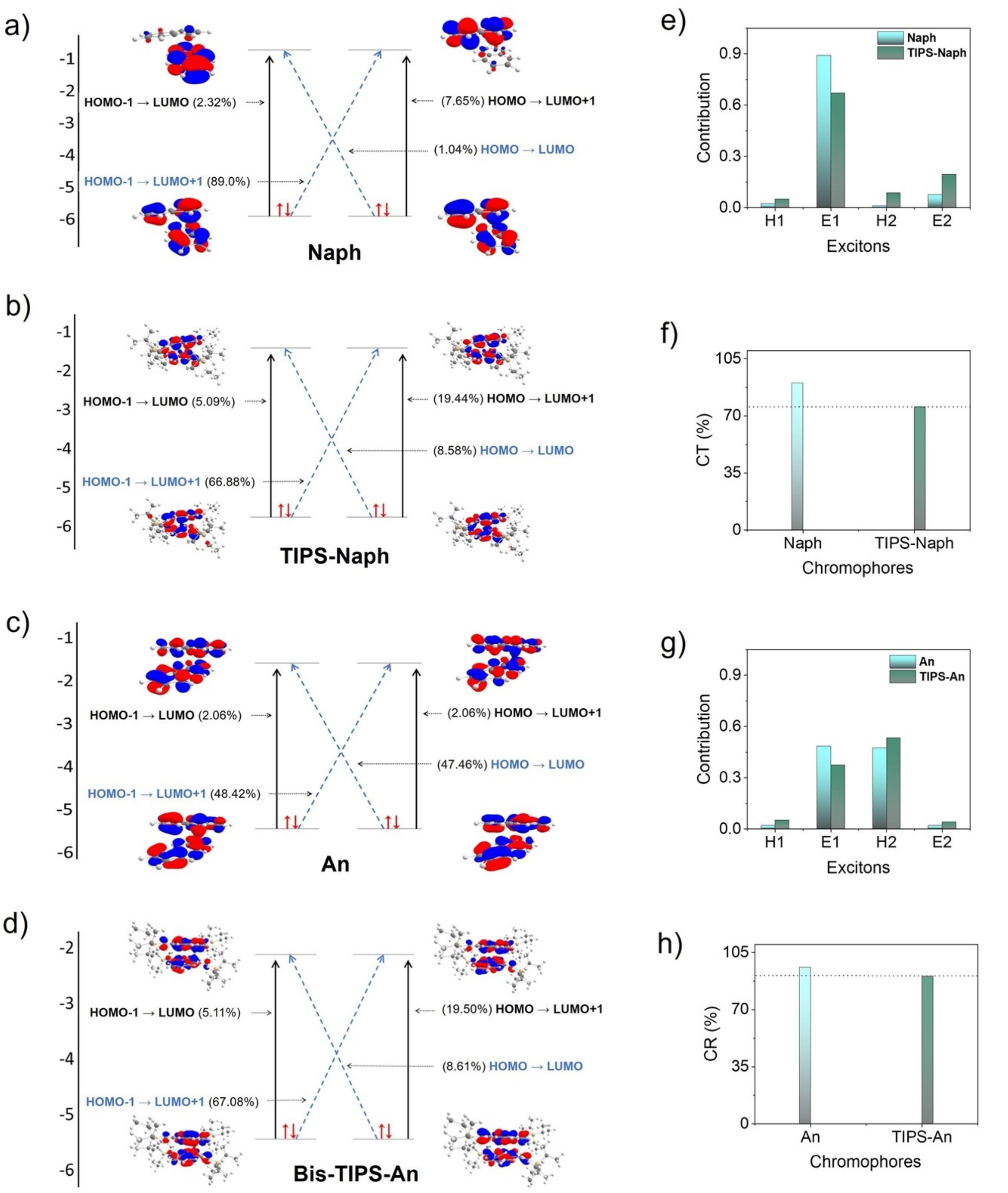

Fig. S13.a-d) Illustration of the contribution of linear combination of orbital replacement in **Naph.**(a), bis-**TIPS-Naph** (b), **An** (c) and bis-**TIPS-An** (d). e-h) Plot showing comparative electron (E), and hole (H) contribution during orbital replacement and percentage of CT or CR calculated from E and H contribution during orbital replacement in **Naph** and **TIPS-Naph** (e, f) and **An** and **TIPS-An** (g, h). Energies in eV.

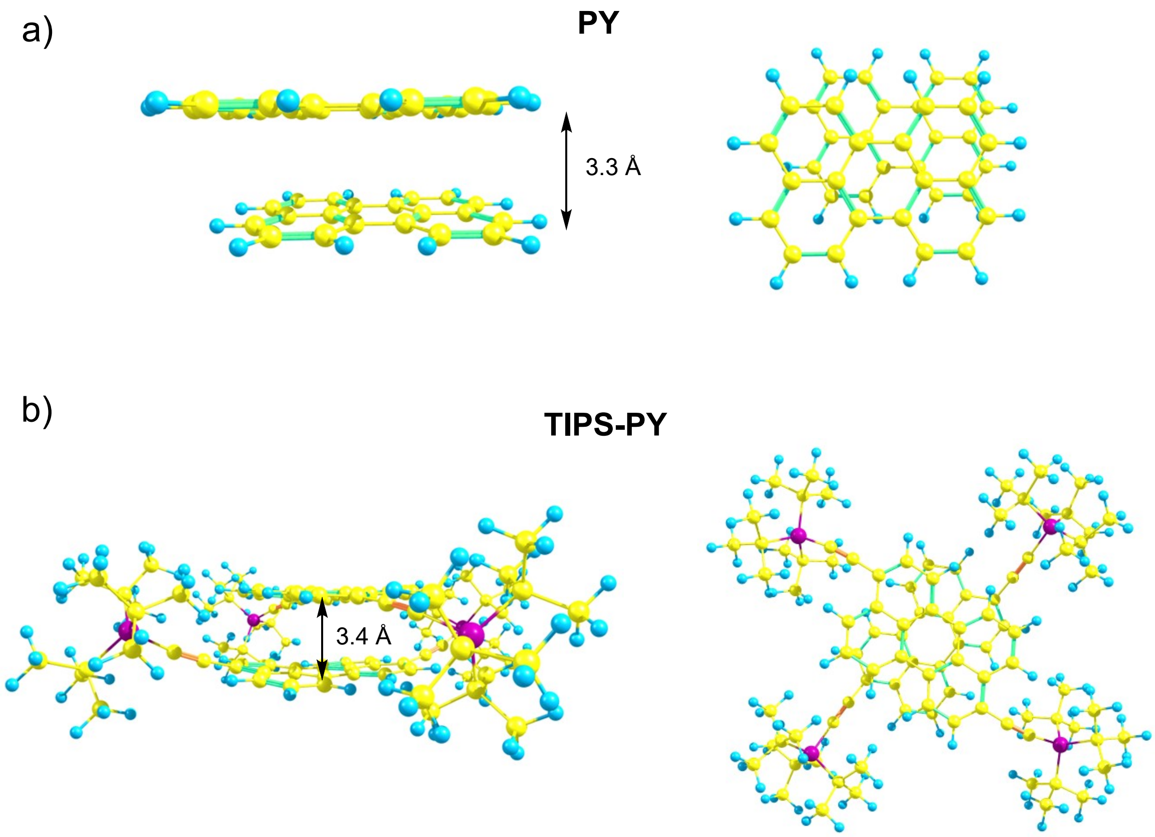

Fig. S14. Two views of the molecular structure of the **PY** and **TIPS-PY** dimers in the ground state (S_0S_0). The dimerization energy for **PY** is -20.43 kcal/mol and for **TIPS-PY** is -31.6 kcal/mol.

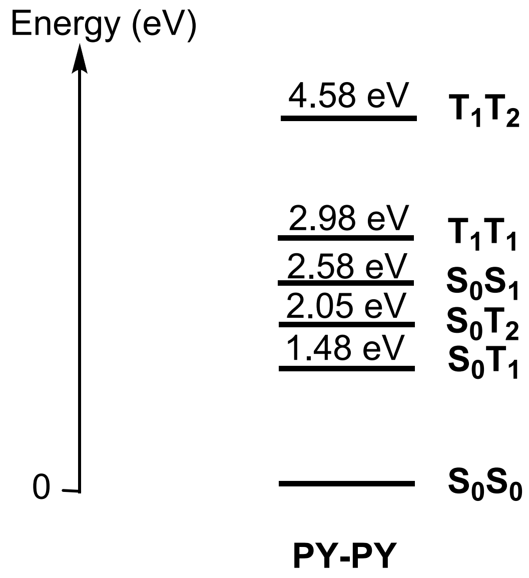

Fig. S15. Calculated excited energy states (energies in eV) for the **PY** dimer. The S_0S_1 state computed at the S_0S_0 geometry, the S_0T_2 state computed at the S_0T_1 geometry and the T_1T_2 state obtained at the T_1T_1 geometry.

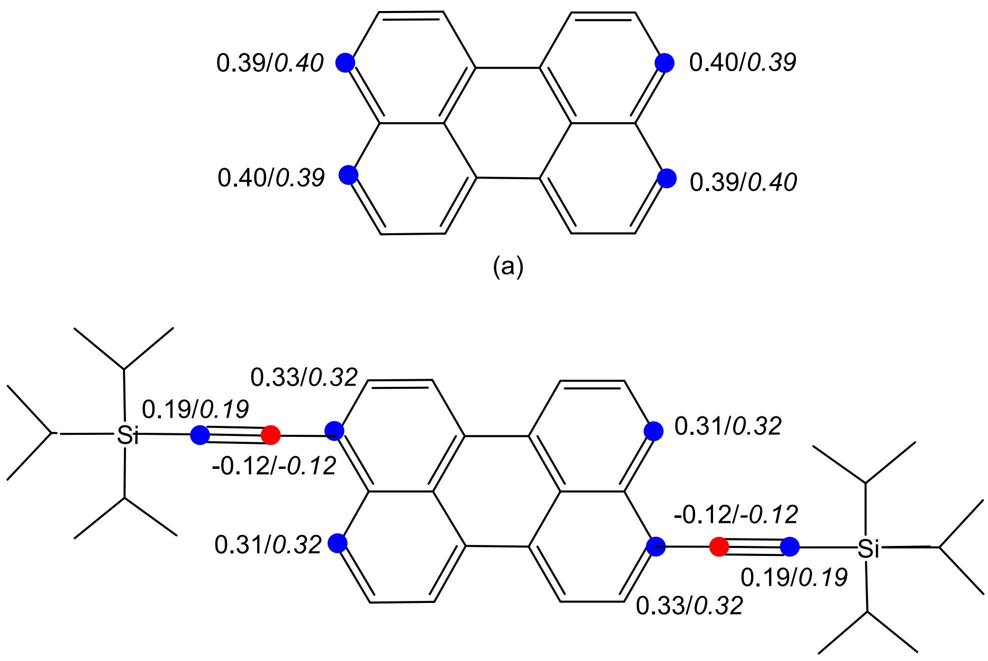

Fig. S16. Calculated spin density distribution (in a.u.) for the T_1T_1 states in the **PY** and **TIPS-PY** dimers. The values in italics correspond to the second unit of the dimer. For **TIPS-PY**, the calculation has been done at the geometry of the S_0S_0 state. Only the values of the spin density of the atoms that change the most from **PY** to **TIPS-PY** are shown.

Table S1. Calculated ΔE_{ST} values with different functionals tested with the 6-311G(d,p) basis set.

**Molecule**	**Functional**	**ΔE_{ST} (eV)**	**%error**
Anthracene	UB3LYP-D3(BJ)	1.82	2.26
	ωB97X	1.90	7.17
	M06-2X	2.42	36.51
	PBE0	1.80	1.51
	**Experimental**[15]	1.85	
9,10-diphenylanthracene	UB3LYP-D3(BJ)	1.73	1.25
	ωB97X	1.79	4.73
	M06-2X	1.94	13.55
	PBE0	1.70	0.43
	**Experimental**[16]	1.77	
9,10-bis[tri-tert-butylsilyl)ethynil]anthracene	UB3LYP-D3(BJ)	1.36	3.30
	ωB97X	1.38	2.26
	M06-2X	2.18	54.36

	PBE0	1.33	5.36
	**Experimental**[17]	1.37	
Perylene	UB3LYP-D3(BJ)	1.50	0.46
	ωB97X	1.67	10.84
	M06-2X	1.80	19.38
	PBE0	1.49	1.38
	**Experimental**[18]	1.53	

Table S2. Calculated excited state energies of **TIPS-An** and **TIPS-Naph** chromophores.

**Compound**	**T_1 (eV)**	**T_2 (eV)**	**$2T_1$-T_2 (meV)**
**TIPS-An**	1.33	3.69	-103
**TIPS-Naph**	2.24	2.62	186

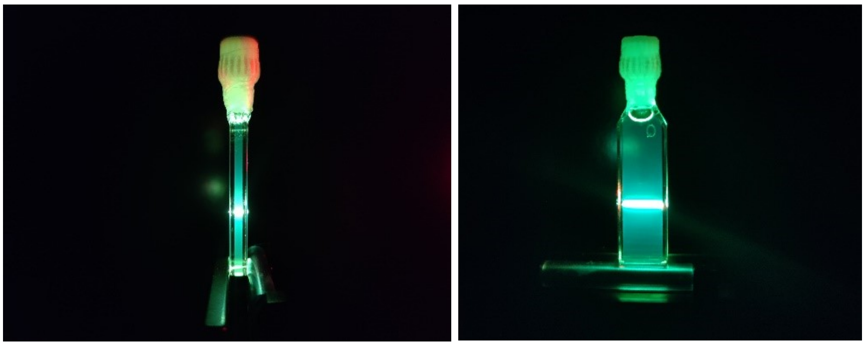

Fig. S17. Digital images showing green UC emission of **TIPS-PY:PdTPBP** solution in THF (λ_{ex} = 640 nm CW laser). TIPS-PY = 1 mM, and PdTPBP = 10 µM

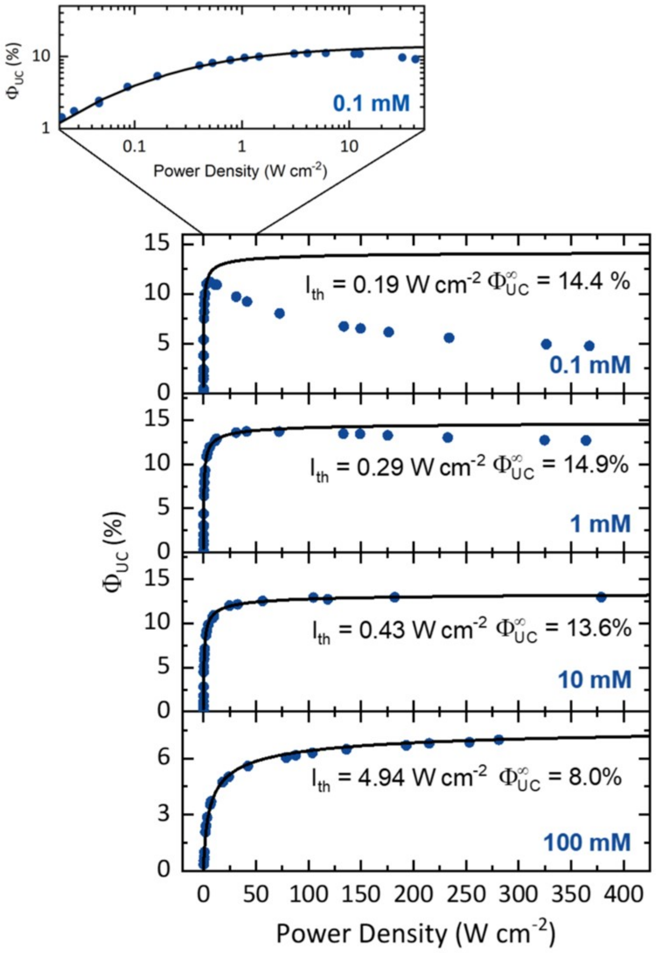

Fig. S18. UC quantum yield dependence on excitation power density of studied **TIPS-PY:PdTPBP** solutions at 0.1 mM, 1 mM, 10 mM and 100 mM annihilator concentrations in THF. UC threshold and maximum quantum yield values indicated. λ_{ex} = 640 nm CW laser. The black lines represent the fit proposed by Murakami et al. (Ref. 44 main manuscript) to estimate UC threshold and maximum UC quantum yield values.

Table. S3. Summary of the calculated anti-stokes shifts at different **TIPS-PY** concentration in THF

TIPS-PY (mM)	PdTPBP (mM)	Lowest absorption maximum of PdTPBP (eV)	Upconversion maximum of TIPS-PY (eV)	Anti-stokes Shift (eV)
0.1	0.01	1.9806	2.5312	0.5506
1	0.01	1.9806	2.5273	0.5467
10	0.01	1.9806	2.3646	0.384
100	0.01	1.9806	2.1896	0.209

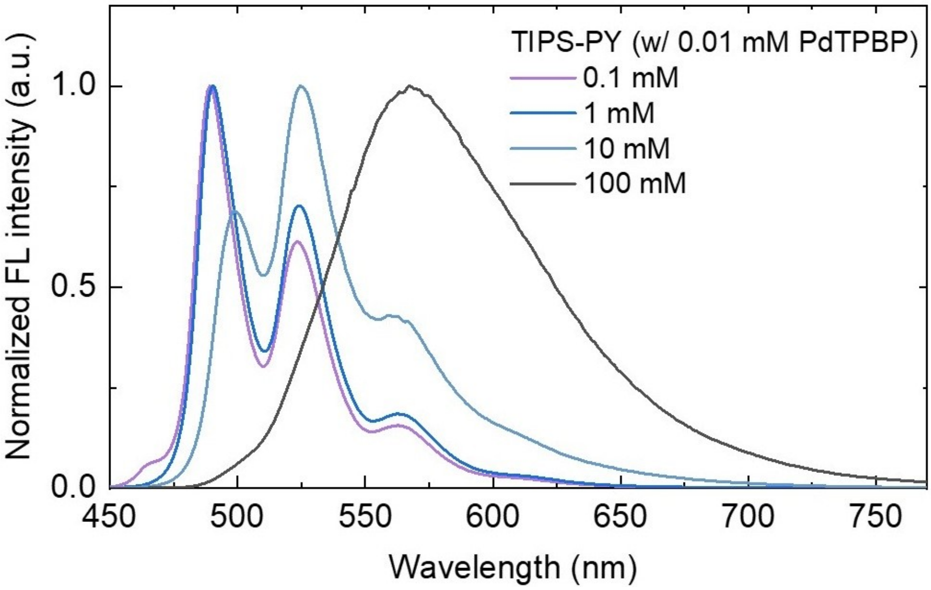

Fig. S19. FL spectra of studied **TIPS-PY:PdTPBP** solutions at 0.1 mM, 1 mM, 10 mM, and 100 mM annihilator concentrations in THF. Solutions excited with 420 nm CW laser.

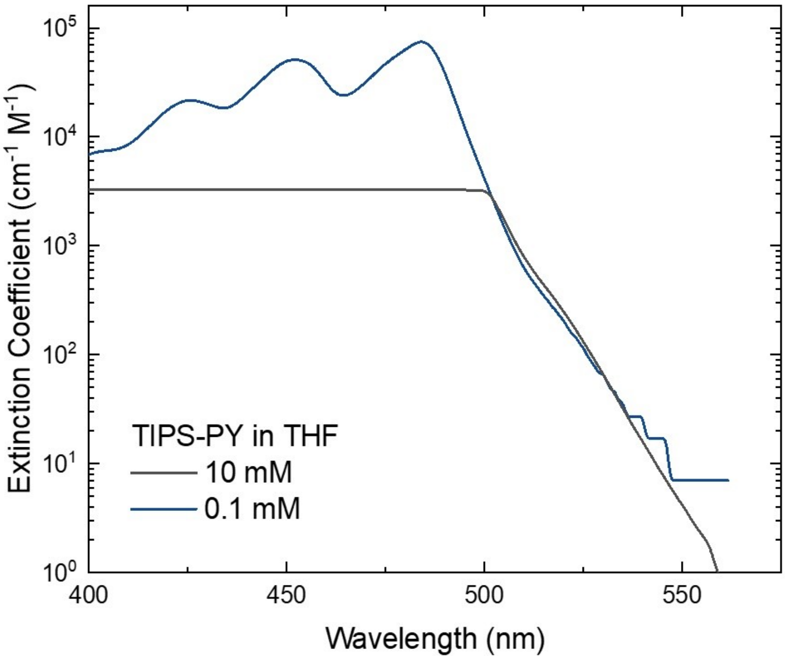

Fig. S20. Absorption spectra tails of **TIPS-PY** at 0.1 mM and 10 mM concentrations in THF.

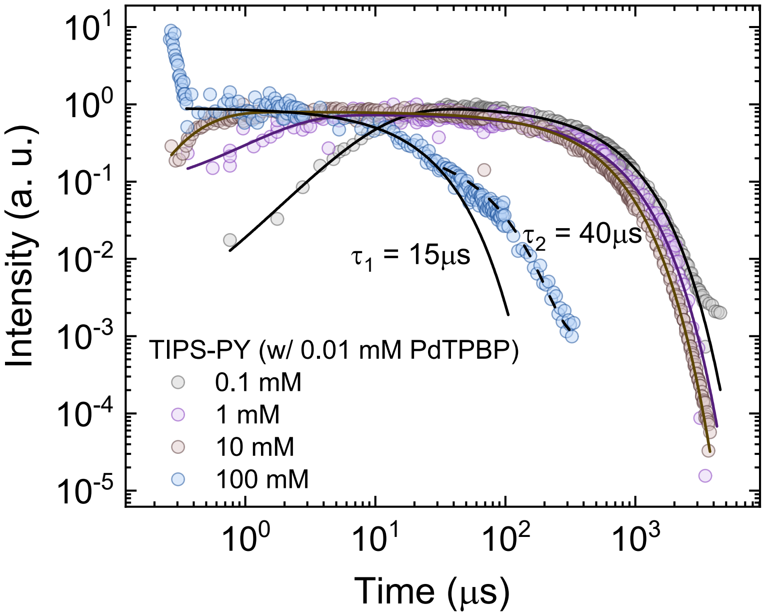

Fig. S21. Transient rise or decay profiles of **TIPS-PY:PdTPBP** system at different annihilator concentrations in deaerated THF solution. All sample solutions were excited with a 640 nm laser at a 1 kHz repetition frequency. The solid lines indicate exponential fits of the rise or decay profiles according to the method reported elsewhere.[19]

Table S4. Rise time and triplet lifetime values of the **TIPS-PY:PdTPBP** system at different annihilator concentrations.

Concentration of **TIPS-PY** (mM)	Rise time (μs)	Triplet lifetime (μs)
0.1	8.16	1250
1	1.55	914
10	0.25	741
100	-	30-80

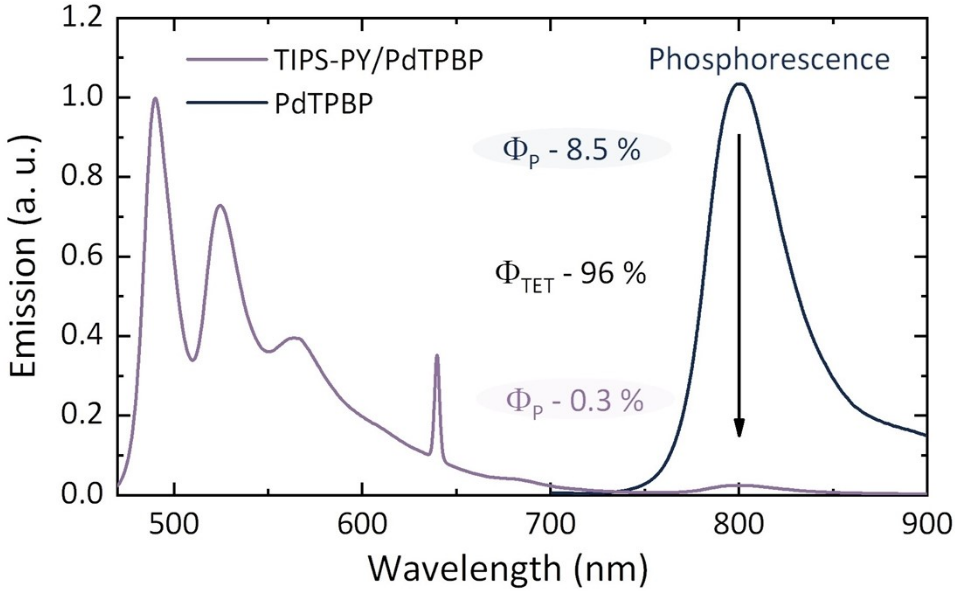

Fig. S22. Phosphorescence spectra of **PdTPBP** in the absence and presence of **TIPS-PY** showing quenching of phosphorescence emission by TIPS-PY t around 800 nm and UC emission in the range of 470-540 nm upon 640 nm CW laser excitation. **TIPS-PY:PdTPBP** (1 mM: 0.01 mM).

Table S5. Summary of various photophysical parameters calculated during TTA in **PY**: **PdTPBP** and **TIPS-PY**: **PdTPBP** solutions upon 640 nm laser excitation in deaerated THF (annihilator = 0.1 mM and sensitizer = 10 µM). [a]Spontaneous triplet exciton decay lifetime. [b]Triplet decay rate. [c]β value estimated from the fits. [d]k_{TTA} of **PY**, obtained from the literature. [e]Calculated initial triplet concentration. [f]Estimated k_{TTA} of **TIPS-PY** assuming $[^3A^*]_0$ value is the same as in **PY** at identical excitation conditions.

**Sample**	Excitation power density (mW cm^{-2})	τ_T [a] (µs)	k_T [b] (s^{-1})	β [c]	k_{TTA} reported[d] (M^{-1} s^{-1})	$[^3A^*]_0$ [e] (M)	k_{TTA} estimated[f] (M^{-1} s^{-1})
**PY**	1.2	542	1.85E+03	0.33	19.0E+08	2.43E-07	
	12	542	1.85E+03	0.74	19.0E+08	1.35E-06	
	24	542	1.85E+03	0.80	19.0E+08	1.97E-06	
	190	542	1.85E+03	0.88	19.0E+08	3.40E-06	
**TIPS-PY**	1.2	1250	8.00E+02	0.23			4.83E+08
	12	1250	8.00E+02	0.59			4.26E+08
	24	1250	8.00E+02	0.74			5.74E+08
	190	1250	8.00E+02	0.84			6.02E+08

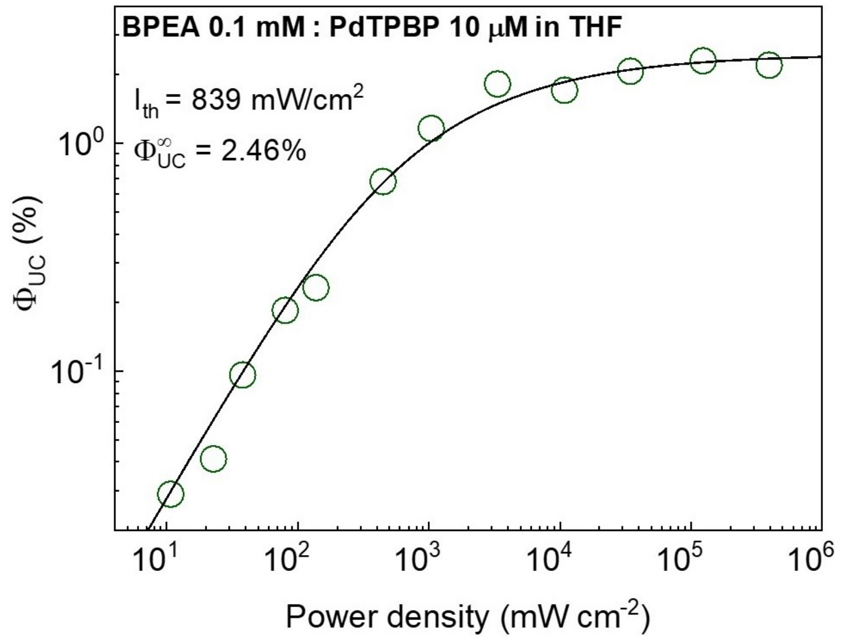

Fig. S23. UC quantum yield dependence on excitation power density of the studied **BPEA:PdTPBP** system in deaerated THF at 0.1 mM and 10 µM concentrations, respectively. UC threshold and maximum quantum yield values indicated. λ_{ex} = 640 nm CW laser. The black lines represent the fit proposed by Murakami et al. (Ref. 44 main manuscript) to estimate UC threshold and maximum UC quantum yield values.

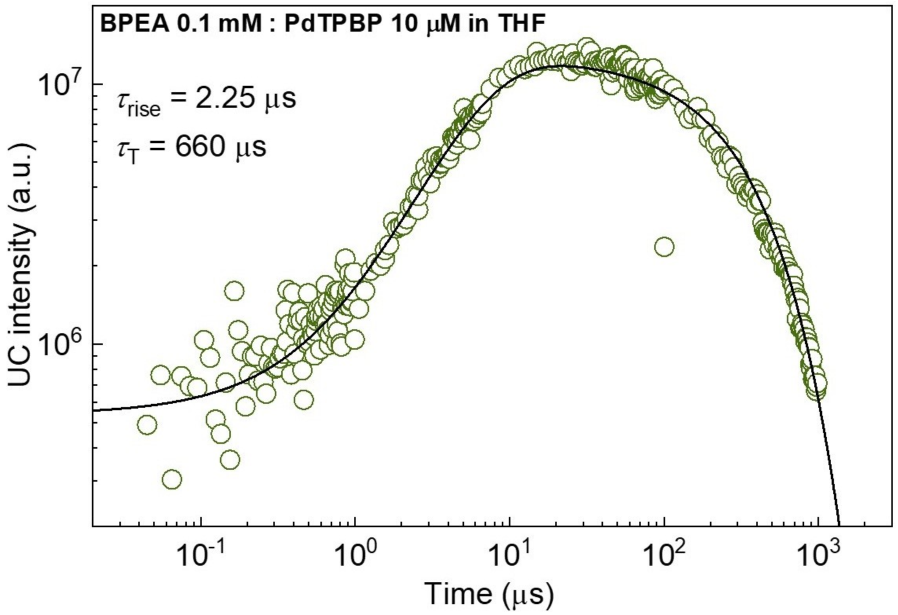

Fig. S24. Transient rise and decay profiles of **BPEA:PdTPBP** system in deaerated THF solution at 0.1 mM and 10 µM concentrations, respectively. The solution was excited with a 640 nm laser at a 1 kHz repetition frequency. The solid line indicates an exponential fit of the rise and decay profile according to the method reported elsewhere.[19]

Table S6 UC parameters of BPEA:PdTPBP UC solution in deaerated THF at 0.1 mM and 10 µM concentrations, respectively.

BPEA	ϕ_{FL}[a], %	ϕ_{UC}[b], %	ϕ_{UC}^{∞}[c], %	ϕ_{TET}[d], %	I_{th}[e], W cm^{-2}	τ_T[f], μs	f[g], %
0.1 mM	81.6	2.20	2.46	95	0.84	660	6.3

[a] FL quantum yield of annihilator in UC solution. [b] reabsorption corrected maximum measured UC quantum yield values [c] maximum attainable UC quantum yield values. [d] TET quantum yield. [e] UC threshold at 38.2% of ϕ_{UC}^{∞}. [f] triplet lifetime ($= 2 \times \tau_{UC}$). [g] statistical probability of singlet generation from two triplets *via* TTA, calculated according to eq. 1.

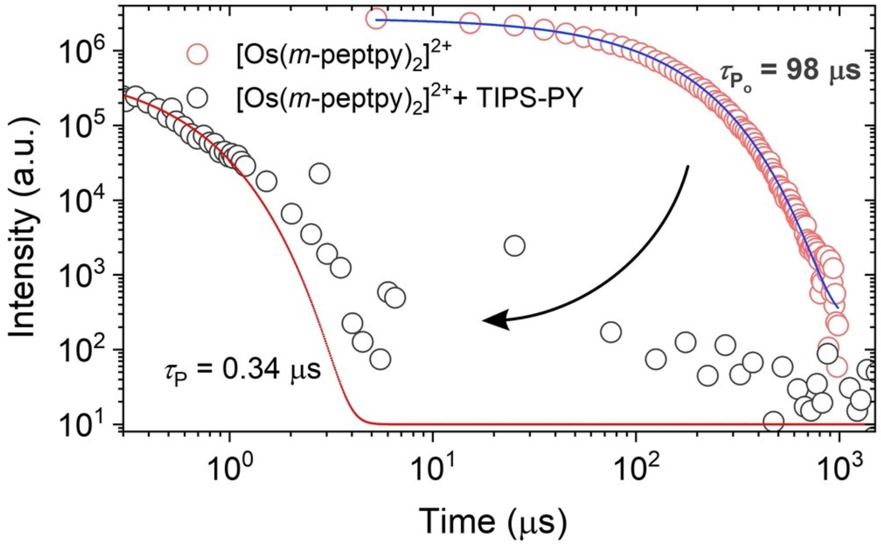

Fig. S25. Phosphorescence transients of **Os(m-peptpy)$_2$(TFSI)$_2$** in deaerated DMF (λ_{em} = 755 nm) in the absence (τ_{Po}) and presence (τ_P) of **TIPS-PY** showing quenching of phosphorescence lifetime due to triplet-energy transfer. **Os(*m*-peptpy)$_2$(TFSI)$_2$** = 0.01 mM and **TIPS-PY** = 1 mM.

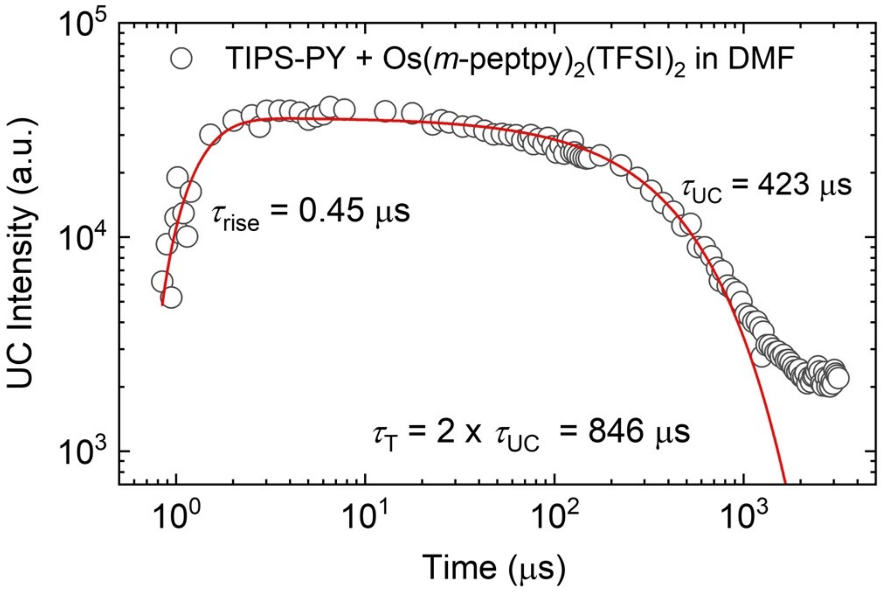

Fig. S26. Upconversion transients of Os(m-peptpy)$_2$(TFSI)$_2$-TIPS-PY system in deaerated DMF showing rise and decay of upconversion emission of TIPS-PY upon 730 nm CW laser excitation. **Os(*m*-peptpy)$_2$(TFSI)$_2$** = 0.01 mM and **TIPS-PY** = 1 mM.

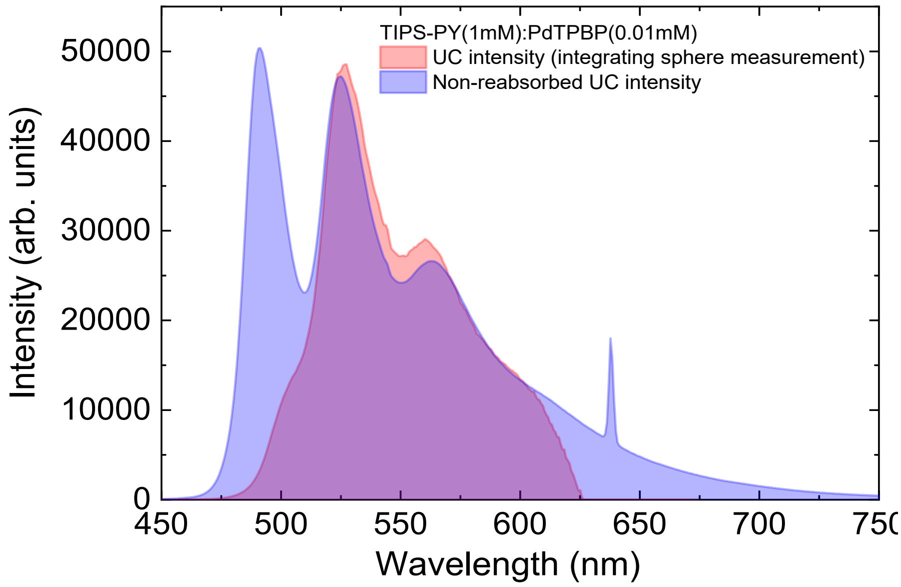

Fig. S27. An exemplary demonstration of UC spectrum correction for **TIPS-PY** (1 mM): **PdTPBP** (0.01 mM). For clarity, the laser excitation band at 640 nm in the reabsorbed UC spectrum was filtered out.

Experimental

Materials

Palladium(II) meso-tetraphenyl porphine (PdTPBP) was purchased from Frontier Scientific. BPEA was purchased from Sigma Aldrich. Os(*m*-peptpy)$_2$(TFSI)$_2$ was synthesized in Kimizuka Lab, according to the previously published procedure.[19] All solvents including $CDCl_3$, THF, DMF, toluene, ethyl acetate and hexane used in this study were purchased from Merck. All organic compounds and catalysts used in the synthesis of **TIPS-PY** including, perylene, N-bromosuccinimide, $Pd(PPh_3)_2Cl_2$, CuI, PPh_3, diisopropylamine, and triisopropylsilylacetylene were purchased from Merck and used as received.

Sample Preparation

All solutions were prepared in THF and DMF solvents. TIPS-PY was dissolved to produce a concentrated stock solution, while a 0.5 mM PdTPBP and 0.38 mM Os(m-peptpy)$_2$(TFSI)$_2$ stock solutions were prepared. The concentrated annihilator stock solution of 1 mM was diluted to 10^{-5} M to perform photophysical measurements. Further on, the 4 upconverting solutions containing 0.1 mM, 1 mM, 10 mM, 100 mM of emitter, and 0.01 mM of sensitizer were prepared for TIPS-PY:PdTPBP system in THF and 1 upconverting solution of TIPS-PY:Os(m-peptpy)$_2$(TFSI)$_2$ (1mM : 0.01M) system was prepared in DMF. One upconverting solution BPEA:PdTPBP (0.1mM : 0.01M) was prepared in THF. All the samples were prepared in the glovebox under a nitrogen atmosphere with O_2 and H_2O concentrations <0.1 ppm. Two-millimetre width cuvettes containing the UC solutions were sealed inside the glovebox. The sealing was essential to protect the UC solutions from triplet quenching by reactive oxygen species.[20]

Optical Techniques

Absorption spectra of the dilute solutions of the TIPS-PY compounds were recorded using a V-770 Jasco UV-vis-NIR spectrophotometer. Fluorescence (FL) of the dilute solutions was excited at 420 nm using a 12-mW semiconductor laser diode (Picoquant). UC measurements were taken with excitation at 640 nm and 730 nm using a 12-mW power continuous-wave semiconductor laser diode (Picoquant). Steady-state FL and UC emission spectra were measured using a back-thinned CCD spectrometer PMA-12 (Hamamatsu). FL and UC quantum yields were estimated by utilizing an integrating sphere[21] (Edinburgh Instruments) coupled to a CCD spectrometer PMA-12 via an optical fiber, with the continuous-wave semiconductor diode laser (Picoquant) employed as the excitation source. Φ_{UC} for reabsorption was corrected by matching the long-wavelength tail of the reabsorbed UC spectrum (measured using an integrating sphere) with that of the non-reabsorbed UC spectrum. The non-reabsorbed spectrum was obtained by focusing the laser beam at the very edge of a two-millimeter-width cuvette containing the UC solution, while collecting the UC emission in the perpendicular direction. The corrected UC spectrum was then used to estimate Φ_{UC} according to Ref. [21] in SI (See Fig. S25). UC lifetimes were estimated from UC transients measured with an ICCD camera (Andor), using a wavelength-tunable nanosecond Nd:YAG laser (Ekspla) as the excitation source. The triplet lifetime was then estimated as $\tau_T = 2 \times \tau_{UC}$. Note also that the UC transients for TIPS-PY concentrations of 0.1, 1, and 10 mM exhibited single-exponential decay profiles. However, at a concentration of 100 mM, due to TIPS-PY aggregation, a dispersion in τ_{UC} (ranging from 15 to 40 µs) was observed. The shorter τ_1 value fit the prompt part of the decay well, while the longer τ_2 fits the later-stage decay. This dispersion implies triplet lifetimes ranging from 30 to 80 µs (see Table 1).

References

1 M. Tracy, S. Singh, *Sony Corp*, **2016**, WO2017197144A1.

2 J.-H. Kim, C. E. Song, I.-N. Kang, W. S. Shind, D-H. Hwang, *Chem. Commun*. **2013**,*49*, 3248-3250.

3 G. M. Sheldrick, *Acta Crystallogr. Sect. C Struct. Chem.* **2015**, *71*, 3–8.

4 G. M. Sheldrick, *Acta Crystallogr. Sect. A Found. Crystallogr.* **2015**, *71*, 3–8.

5 O. V. Dolomanov, L. J. Bourhis, R. J. Gildea, J. A. K. Howard, H. Puschmann, *J. Appl. Crystallogr.* **2009**, *42*, 339–341.

6 C. Adamo, V. Barone, *J Chem Phys* **1999**, *110*, 6158–6170.

7 S. Grimme, J. Antony, S. Ehrlich, H. Krieg, *J Chem. Phys.* **2010**, *132*, 154104.

8 E. R. Johnson, A. D. Becke, *J Chem. Phys.* **2005**, *123*, 024101.

9 A. D. Becke, E. R. Johnson, *J. Chem. Phys.* **2005**, *123*, 154101

10 R. Krishnan, J. S. Binkley, R. Seeger, J. A. Pople, *J. Chem. Phys.* **1980**, *72*, 650–654.

11 M. J. Frisch, G. W. Trucks, H. B. Schlegel, G. E. Scuseria, M. A. Robb, J. R. Cheeseman, G. Scalmani, V. Barone, G. A. Petersson, H. Nakatsuji, X. Li, M. Caricato, A. V Marenich, J. Bloino, B. G. Janesko, R. Gomperts, B. Mennucci, H. P. Hratchian, J. V Ortiz, A. F. Izmaylov, J. L. Sonnenberg, D. Williams-Young, F. Ding, F. Lipparini, F. Egidi, J. Goings, B. Peng, A. Petrone, T. Henderson, D. Ranasinghe, V. G. Zakrzewski, J. Gao, N. Rega, G. Zheng, W. Liang, M. Hada, M. Ehara, K. Toyota, R. Fukuda, J. Hasegawa, M. Ishida, T. Nakajima, Y. Honda, O. Kitao, H. Nakai, T. Vreven, K. Throssell, J. A. Montgomery Jr., J. E. Peralta, F. Ogliaro, M. J. Bearpark, J. J. Heyd, E. N. Brothers, K. N. Kudin, V. N. Staroverov, T. A. Keith, R. Kobayashi, J. Normand, K. Raghavachari, A. P. Rendell, J. C. Burant, S. S. Iyengar, J. Tomasi, M. Cossi, J. M. Millam, M. Klene, C. Adamo, R. Cammi, J. W. Ochterski, R. L. Martin, K. Morokuma, O. Farkas, J. B. Foresman, D. J. Fox, *Wallingford, CT* **2016**.

12 A. D. Becke, *J. Chem. Phys.* **1993**, *98*, 5648–5652.

13 C. Lee, W. Yang, R. G. Parr, *Phys. Rev. B* **1988**, *37*, 785.

14 F. Plasser, *J. Chem. Phys.* **2020**, *152*, 084108.

15 M. R. Padhye, S. P. McGlynn, M. Kasha, *J. Chem. Phys.* **1956**, 24, 588–594.

16 J.S. Brinen, J.G. Koren, *Chem. Phys. Lett*. **1968**, 2, 671-672.

17 J. K. H. Pun, J. K. Gallaher, L. Frazer, S. K. K. Prasad, C. B. Dover, R. W. MacQueen and T. W. Schmidt, *J. Photonics Energy*, **2018**, 8, 1

18 R. H. Clarke, R. M. Hochstrasser, *J. Mol. Spectrosc*. **1969**, 32, 309-319.

19 E. M. Gholizadeh, S. K. K. Prasad, Z. L. Teh, T. Ishwara, S. Norman, A. J. Petty, J. H. Cole, S. Cheong, R. D. Tilley, J. E. Anthony, S. Huang, T. W. Schmidt, *Nat. Photonics* **2020**, *14*, 585–590.

20 T. N. Singh-Rachford, F. N. Castellano, *J. Phys. Chem. A* **2008**, *112*, 3550–3556.

21 J. C. De Mello, H. F. Wittmann, R. H. Friend, *Adv. Mater.* **1997**, *9*, 230–232.
